# Nonlinear Mechanical Effect of Free Water on the Dynamic Compressive Strength and Fracture of High-Strength Concrete

**DOI:** 10.3390/ma14144011

**Published:** 2021-07-18

**Authors:** Evgeny V. Shilko, Igor S. Konovalenko, Ivan S. Konovalenko

**Affiliations:** 1 Institute of Strength Physics and Materials Science of Siberian Branch Russian Academy of Sciences (ISPMS SB RAS), 2/4, pr. Akademicheskii, 634055 Tomsk, Russia; 2 School of Core Engineering Education, National Research Tomsk Polytechnic University, 30, Lenin Avenue, 634050 Tomsk, Russia; ivkon@tpu.ru

**Keywords:** water-saturated concrete, two-scale porosity, permeability, fluid filtration, dynamic loading, fracture, compressive strength, computer simulation, discrete element method, coupled poroelastic model

## Abstract

It is well-known that the effect of interstitial fluid on the fracture pattern and strength of saturated high-strength concrete is determined by qualitatively different mechanisms at quasi-static and high strain rate loading. This paper shows that the intermediate range of strain rates (10^−4^ s^−1^ < $\dot{\varepsilon }$ < 10^0^ s^−1^) is also characterized by the presence of a peculiar mechanism of interstitial water effect on the concrete fracture and compressive strength. Using computer simulations, we have shown that such a mechanism is the competition of two oppositely directed processes: deformation of the pore space, which leads to an increase in pore pressure; and pore fluid flow. The balance of these processes can be effectively characterized by the Darcy number, which generalizes the notion of strain rate to fluid-saturated material. We have found that the dependence of the compressive strength of high-strength concrete on the Darcy number is a decreasing sigmoid function. The parameters of this function are determined by both low-scale (capillary) and large-scale (microscopic) pore subsystems in a concrete matrix. The capillary pore network determines the phenomenon of strain-rate sensitivity of fluid-saturated concrete and logistic form of the dependence of compressive strength on strain rate. Microporosity controls the actual boundary of the quasi-static loading regime for fluid-saturated samples and determines localized fracture patterns. The results of the study are relevant to the design of special-purpose concretes, as well as the assessment of the limits of safe impacts on concrete structural elements.

## 1. Introduction

High-strength concrete is often used as a high-performance building material for structures and structural elements operating in aquatic or water-saturated environments [1,2,3,4,5]. Well-known examples are dams, bridge piers, offshore platforms, quay walls, mine shaft shells in flooded soils, etc. Since concrete is a permeable porous material, the pore space of mortar (the main component of concrete) is gradually filled with water. In particular, the laboratory study [6] showed that samples of mortar 10 cm thick are saturated with water up to 80% within the first day of being in the vessel. This estimate is confirmed by other experimental studies, which indicate that close to full saturation of laboratory concrete samples with water or aqueous solution is achieved within 1–3 days [7,8,9]. The surface layers of massive concrete structures in a water or water-saturated environment can therefore be completely saturated with water to a depth of at least 10^1^ cm and higher.

Water or an aqueous solution in the pore space has a significant effect on the mechanical properties of concrete. The pore fluid is reported to be responsible for numerous phenomena of premature fracture of various brittle materials (including concretes) under loads significantly lower than permissible for these materials in a dry state [6,10,11,12,13,14,15,16,17].

At low strain rates corresponding to a quasi-static loading mode, the pore fluid typically has a negative effect on the strength of concrete. This effect most clearly appears under compression, which is the typical type of stress state of elements of concrete constructions. Different experimental studies show that the compressive strength of concrete samples can decrease by 10–20 percent with an increase in water saturation [6,7,10,18,19,20]. Moreover, compression tests on water-saturated samples in a high water pressure device (at different values of water pressure) demonstrate an additional decrease in quasi-static strength by more than 10% with an increase in pore pressure from atmospheric value to several MPa [7]. These results are consistent with many earlier studies on various types of concrete including studies at higher water and confining pressures [21]. Note that a significant decrease in quasi-static strength is achieved in these cases even despite an increase in specimen confinement (lateral pressure increases).

The key factor determining the described decrease in strength is pore pressure (pressure of interstitial water) [18,20]. Various authors propose different mechanisms of the effect of pore pressure on the strength of the material at low strain rates, but they are all associated with the growth of tensile volumetric stress in solid skeleton due to the compression of the pore space and pore water. Expanding effect of water pressure causes local splitting formation, acceleration of existing and newly formed cracks and finally promotes macroscopic fracture at lower applied loads [21,22]. Note also that the mechanical effect of the interstitial fluid pressure on the compressive strength of porous materials with a brittle skeleton (including concrete) becomes significant even at low porosity of the order of a few percent [23].

Due to the connectivity of the pore space of concrete, the rate of pore fluid pressure growth during compression depends on the rate of loading. Indeed, the key physical property of water that qualitatively distinguishes it from a solid-phase skeleton is mobility (fluidity). An increase in pore pressure in a sample under compression leads to a redistribution of free (chemically unbound) water in a system of interconnected material discontinuities (pores, channels, cracks). Fluid flow provides redistribution and balancing of local stresses in a fluid-saturated material. Moreover, the difference in fluid pressure between the central part of the compressed sample and the outer surfaces causes the outflow of “excess” pore fluid and a decrease in the overall stress level. It is clear that the magnitude of the pore pressure gradients for a particular concrete is determined by the strain rate (that is, the rate of compression of the pore space). Therefore, the mechanical effect of interstitial water pressure on the stress state and mechanical characteristics of concrete as a porous material should depend on the ratio of the rate of skeleton straining (characterized by applied strain rate $\dot{\varepsilon }\sim 1/T_{def}$, where *T_def_* is the timescale of skeleton and pore space deformation) to the pore fluid flow rate (characterized by Darcy timescale *T_Darcy_*) [24,25].

There are two “limiting” ranges of the control parameter $\dot{\varepsilon }$ in relation to pore fluid flow rate in the pore space. The first of these is related to quasi-static loading conditions ($\dot{\varepsilon }$ < 10^−4^ s^−1^ for typical water-saturated concrete materials). Within this strain rate interval, the pore fluid has time to redistribute in the sample (and outflow). Therefore, the fluid flow compensates for the compression of the pore space, and the pore pressure slowly increases during loading. Mechanical characteristics of water-saturated concrete gradually tend to that of dry samples with the loading decrease rate [21]. The second “limiting” interval covers “large” strain rates ($\dot{\varepsilon }$ > 10^1^ s^−1^). In this strain rate interval, the flow of pore fluid is negligible due to the inertia of the flow process, and the pore pressure increase rate tends to a maximum. However, the expected maximum negative effect of the pore fluid on the strength of concrete does not occur, since the dynamic (inertial) properties of the solid-phase skeleton and the viscous properties of the pore fluid play a leading role here. In particular, the incubation time of damages and cracks in the solid-phase skeleton of concrete at high strain rates becomes comparable (in order of magnitude) with the characteristic loading time [11,26]. The finite rate of crack growth provides a significant increase in the dynamic strength of concrete (specific value of the ultimate load) with an increase in $\dot{\varepsilon }$ [6,10,27,28,29,30,31,32,33]. For example, at $\dot{\varepsilon }$~10^2^ s^−1^ the DIF (dynamic increase factor) value approaches 2 [6,28,31]. Pore water is a viscous liquid, and the forces of its viscous resistance to crack formation (they are attributed to meniscus [17,22,34], Stefan [10,35,36], and Newton inner friction [18,22] forces) exceed the negative effect of high pore pressure at $\dot{\varepsilon }$ > 10^1^ s^−1^. These viscous forces are responsible for a strong positive influence of pore fluid on dynamic strength at high strain rates. This effect is physically interpreted as an increase in fracture incubation time [37].

The vast majority of studies are focused on studying fracture patterns and determining the strength characteristics of water-saturated concretes at the two described “limiting” intervals of strain rate. At the same time, they are connected by a wide but much less studied “intermediate” interval (10^−4^ s^−1^ < $\dot{\varepsilon }$ < 10^1^ s^−1^). The mechanical behavior of water-saturated concretes and the strain rate dependence of strength within this interval have distinctive features. In this “intermediate” region, the rate of growth of pore pressure during loading becomes comparable and exceeds the characteristic rates of filtration of pore fluid but the role of inertia and viscous factors are still small or even negligible. Therefore, fracture pattern and compressive strength of water-saturated concrete are largely determined by a dynamic factor characterizing the balance of the rates of two competing processes: applied deformation (pore compression) and pore fluid flow.

Despite the large number of works devoted to the study of the mechanical effect of pore water, there are still no works with a systematic analysis and generalization of the effect of the competition of two counter-directed processes on fracture behavior and compressive strength of high-strength concretes in the “intermediate” interval of strain rates (10^−4^ s^−1^ < $\dot{\varepsilon }$ < 10^1^ s^−1^). The relevance of such an analysis is particularly determined by the fact that many dynamic impacts on underwater structures during operation (including multiple impacts of heavy floating objects [38,39,40]) occur at strain rates lying in this interval. The study and generalization of the mechanical effect of pore water on the features of concrete fracture and dynamic strength at strain rates above quasi-static and below high rate intervals is a topic that has both fundamental significance and practical implications for the assessment of the limits of safe impacts on saturated structural elements and the optimal design of the internal structure of concrete.

Experimental studies are able to provide comprehensive information about the nature and characteristics of the macroscopic fracture of concrete samples and their strength parameters. At the same time, the possibilities of laboratory experiments for analyzing the nucleation and evolution of mesoscale damages and cracks, the role of the interaction of constituent phases, and their contributions to fracture are limited. Mesoscale computer modeling is an efficient tool for such research. The key advantage of virtual (computer) study is the ability to qualitatively and quantitatively determine the contributions of the structural parameters and the pore fluid to the change in the fracture pattern and dynamic strength of concrete samples.

The traditional way is to use a powerful continuum-based finite element method (FEM) with the accurate implementation of mesoscale geometries, nonlinear material models including the behavior of interface zones, and pore fluid infiltration. Thilakarathna et al. [41] developed a mesoscopic FEM model of concrete, which takes into account complex shapes of aggregates and accurate material models for mortar, aggregates, and interphase boundaries. They modeled mortar as elastic-plastic material and applied non-associated plastic flow law with Lubliner yield criterion and Drucker–Prager hyperbolic potential function. Fracture of mortar was considered gradual softening after ultimate stress was achieved. Aggregates were considered elastic-brittle with a pressure-dependent fracture criterion (the same approximation for hardening inclusions is used in most models of concrete). Mazzucco et al. [42] proposed a method of constructing a robust mesoscale FEM model based on X-ray Computed Tomography. They used non-associated plastic flow law with Menetrey-William yield function and Grassl potential function. Failure was modeled as material gradual degradation controlled by a special isotropic damage parameter. A similar (Menetrey-William-based) fracture-plastic continuous model was used by Sucharda et al. [43] and Valikhani et al. [44] to describe inelastic behavior and continuous degradation of normal and ultra-high performance concretes. Niknezhad [45] used an approach similar to [41], with the Kachanov-Rabotnov damage plasticity constitutive model and non-associated plastic flow law. Interfaces were modeled in all the abovementioned papers using the model of zero thickness cohesive zone with piecewise linear (or sometimes nonlinear) traction-separation law. The numerical values of the parameters of plasticity and post-peak behavior models are usually determined on the basis of macroscopic experimental data for concrete components and by trial and error for interfaces. An extension of these “classical” quasistatic models is a multiscale model by Vorel et al. [46], where the mechanical properties of the matrix are derived from microscale modeling. We want to particularly mention the dynamic model of water-saturate concrete by Huang et al. [47,48], which applies the Hugoniot diagram to derive the equation of state of concrete within the wide range of pressure values and takes into account pore pressure using the Hugoniot mixing rule or effective stress law (Biot’s linear model of poroelasticity). The developed FEM models provide great opportunities for analyzing the localization of inelastic strains and degradation of individual components and the whole sample, accurate reproduction and prediction of diagrams of monotonic and cyclic loading, studying the effect of pore fluid on the acoustic and elastic characteristics of fluid-saturated material, and predicting the changes in transport properties (permeability, porosity) of concrete due to the accumulation of damages. Despite the advantages of the continuum-based approach, its application to simulate damage localization including crack weaving and branching, and the resulting size-effect meets well-known difficulties. As was mentioned above, the fracture surfaces are usually represented using approximations or models, which smear real discontinuity [41,42,43,44,45,49,50] or embed it (extended implementation of FEM [51]).

An attractive alternative is the use of particle-based (“discrete”) numerical methods. These methods are based on representing material as an ensemble of finite-sized particles that can change the state of bonding with neighbors (bonded-to-unbonded transition) and hence change the neighborhood. The formalism of these methods is therefore not limited by continuity constraints. The key advantage of discrete methods is the inherent ability to model discontinuity formation as an extremely localized process by means of separating the surfaces of adjacent particles. Several discrete methods deserve special attention in the context of an adequate description of the failure of concretes and other brittle porous materials in the range of scales. The Lattice Discrete Particle Model (LDPM) developed by G. Cusatis et al. represents concrete as an ensemble of polyhedral aggregates implemented in a matrix as a binding medium [52,53] and connected through a 3D lattice. Input model parameters are typical material parameters including elastic moduli, compressive and shear strengths, parameters of the post-peak softening curve, friction coefficient, etc. The values of these parameters for the particular concrete are taken from experiments and trial and error procedures. The developed formalism makes it possible to effectively take into account the accumulation of low-scale damages, explicitly model mesoscopic fracture through breaking the bond (springs) between adjacent particles, and implement the strain rate dependence of crack cohesion [54,55]. Note that an alternative dynamic fracture model based on the concept of kinetic strength theory was also implemented within the spring network formalism [56]. The developed LDPM models make it possible to reproduce the stress–strain curve of simulated concrete or rock, to analyze mesoscopic damage accumulation and crack initiation and development as well as to explicitly obtain dilatancy/compaction and changes in the porosity and permeability of the material under loading. Advanced LDPM implementations for mesoscopic modeling of the behavior of permeable brittle materials with interstitial fluid include several hybrid techniques [57,58] as well as coupled SPH-LDPM methods [59]. Hybrid techniques allow us to take into account the interconnected low-scale discontinuities, while coupled method makes it possible to explicitly investigate fluid flow through mesoscopic discontinuities. An ideologically similar discrete method used for mesoscale modeling of the mechanical behavior of porous materials (including fracture) and flow of interstitial fluid is the discrete element method (DEM) [60,61]. The key difference between DEM and LDPM as applied to mesocomposite materials (including concrete) is the explicit modeling of all components with an ensemble of particles. The most popular DEM implementation is based on “dividing” the modeled body into equiaxial polygonal regions (discrete elements) and their simplified representation by equivalent balls. Note that this simplification of the element shape is applied only when solving the equations of motion and dividing the vector of element–element interaction force into two formally unrelated components. The ability of DEM to analyze deformation and fracture processes in permeable fluid-saturated brittle materials is similar to LDPM. An additional advantage of DEM is the ability to explicitly model the accumulation of damages in the cement matrix and at the interfaces and evolution of these damages into mesocracks. An important limitation of the classical DEM formalism (as well as the LDPM formalism) is the approximation of rigid particles. This approximation makes it difficult to reproduce the inelastic behavior due to the accumulation and development of lower-scale discontinuities and to model coupled solid-fluid problems for permeable materials with multi-scale porosity. While mesoscale voids with fluid can be modeled explicitly in the classical DEM implementations with rigid elements, low-scale discontinuities (with respect to the modeled mesoscopic scale) with interstitial fluid can be adequately taken into account using only the advanced approximation of deformable particles.

The present paper is devoted to the study of the features of dynamic fracture and strain rate dependence of compressive strength of fluid-saturated high-strength concrete through computer simulation of uniaxial compression of mesoscale samples. The purpose of this investigation is to reveal and generalize the regularities of change in fracture pattern (including mesoscopic mechanisms) and compressive strength of fluid-saturated high-strength concrete within the practically important “intermediate” interval of strain rates (10^−4^ s^−1^ < $\dot{\varepsilon }$ < 10^1^ s^−1^) between quasi-static and high rate loading ranges.

The numerical study was carried out with the use of the advanced discrete element technique, namely the method of homogeneously deformable discrete elements [62,63]. For adequate modeling of the two-scale pore structure of high-strength concrete with interstitial fluid, we implemented the coupled mechanical model of permeable brittle solids. The model takes into account elastic-plastic deformation of the skeleton, the flow of fluid in the pore space (including redistribution between two pore subsystems of different scales), and the mutual influence of these processes (through deformation of the pore space and the corresponding change in pore fluid pressure) [64].

The remainder of the paper is organized as follows. Section 2 explains the keystones of the physical and numerical (discrete-element) mesoscopic model of fluid-saturated high-strength concrete including the details of the implementation of non-associated plastic flaw law for cement matrix, the two-parameter criterion of local fracture, and mechanical interaction of concrete with interstitial fluid. Section 3 describes and interprets the dependence of dynamic compressive strength of mesoscopic fluid-saturated concrete samples on the strain rate, the evolution and features of fracture of the samples at different strain rates as well as the role of fluid in low-scale pore network and large-scale pores. We are the first to show that the obtained regularities of the dependence of the strength and fracture can be characterized in terms of a recently proposed dynamic parameter [65], which generalizes the notion of strain rate to fluid-saturated materials and characterizes the balance of processes of skeleton deformation and pore fluid flow. Section 4 discusses the obtained regularities for compressive strength and fracture patterns and shows the contributions of mesoscopic structural elements to the fracture of the samples. The paper ends with the concluding remarks.

## 2. Materials and Methods

### 2.1. The Model of a Mesoscopic Representative Volume of Concrete

We considered mesoscale samples of high-strength concrete with the pore space completely saturated with fluid (water was considered as the “base” fluid). The initial value of the pore pressure (before loading) was assumed to be equal to atmospheric pressure. The mesoscale here means the characteristic scale of the key elements of the internal structure. These are aggregates of millimeter linear dimensions and “micropores” with characteristic dimensions of hundreds of micrometers. The mesoscopic structure of concrete consists of a cement stone (mortar) matrix with large hardening aggregates (particles of basalt crushed stone). The typical form of aggregate particles is equiaxed, the average size of the inclusions is 10–15 mm.

Porosity is among the key structural parameters of cement stone as it largely influences the effective strength and elastic properties. Typical high-strength concrete is characterized by a two-scale pore structure (capillary pores and micropores). The capillary porosity of the cement stone reaches ~6 vol.%, while the volume fraction of micropores is up to 4% [66,67,68]. The characteristic size of micropores is hundreds of micrometers and the micropore shape is close to round. The system of interconnected capillaries is open and determines the permeability of the cement stone. The transverse dimensions of the capillary pore channels can vary over a wide range from 10^−2^ µm to 10^1^ µm. This is determined by various factors including the conditions for the preparation and hardening of concrete as well as the water-to-cement ratio. A decrease in the characteristic transverse size of capillary channels is a consequence of the deposition of cement hydration products in the pore channels during concrete hardening. Hydration product deposition can lead to multiple narrowing of capillary characteristic opening and a decrease in cement permeability by several orders of magnitude [68,69,70]. The connectivity of the capillary pore system with micropores also deteriorates in this case. Difficulty in the redistribution of pore fluid between two pore subsystems can also be a factor affecting the compressive strength and strain rate sensitivity of concrete.

**Figure 1:**
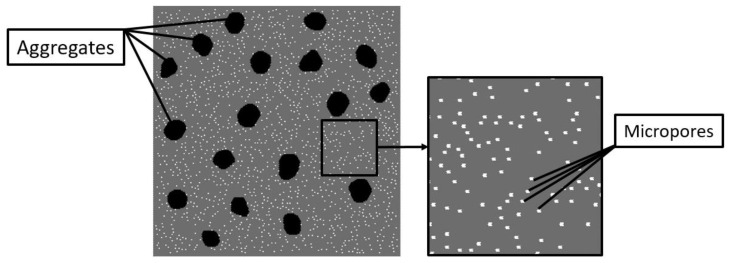
An example of the structure of a mesoscale concrete sample with 10 vol.% of basalt aggregates. The volume fraction of micropores is 4%.

These principal features of the multiscale structure were taken into account in a developed two-dimensional computer model of a representative mesoscale volume of high-strength concrete. The model includes two spatial scales: mesoscopic and microscopic. The mesoscale components of the internal structure of concrete (cement stone, aggregates, micropores) are specified explicitly (Figure 1).

Spatial placement and determination of the geometry of particular aggregates in the model samples were carried out using the Voronoi tessellation algorithm [71]. The volume fraction of basalt aggregates was assumed to be 10% in the present study. The aggregates were approximately equiaxial and angular. Micropores were randomly distributed in the volume of the cement stone matrix. Each micropore was introduced by removing the corresponding area of the material from the initial (non-porous) matrix. We considered mesoscale samples with different volume fractions of micropores ranging from 0% (mortar contains only capillary pores) to 4%. The microporosity of the cement stone was considered mono-sized, the shape of the micropores was round (the diameter of the micropore is 500 μm).

The fluid in the micropores of the cement stone was described effectively. Each micropore was characterized by the mass, density, and pressure of the liquid in its volume (we used the approximation of the equipartition of the liquid density in the micropore). The volume of a micropore changes during loading. The current value of a micropore volume during the simulation is calculated using the special algorithm described in [63].

The internal pore structure of the cement stone at lower scales was taken into account effectively. It was characterized by capillary porosity and permeability. Capillary permeability of the cement stone *k* was assumed to be linearly related to the capillary porosity φ (φ = 6% in this study) and the characteristic transverse size (effective diameter) of the capillary pore channels *d_ch_* by the known relation: *k* = φ*d_ch_*^2^ [72]. We varied the effective diameter of the capillary channels *d_ch_* in the range from 0.1 to 1 μm to analyze the effect of the degree of deposition of cement hydration products in the capillary pore space on the dynamic mechanical behavior of fluid-saturated concrete. Capillary porosity was assumed to be connected with micropores.

The fluid in the volume of capillary pores was taken into account effectively and was characterized by local density and pore pressure.

The mechanical characteristics of water were used for pore fluid: density at atmospheric conditions $\rho_{fl}^{0}$ = 1000 kg/m^3^, bulk modulus *K_fl_* = 2.2 GPa.

Basalt was considered an isotropic elastic-brittle material. The cement stone was assumed to be an isotropic elastic-plastic material (the value of inelastic strain is small and comparable to the value of elastic strain). We use the continuum model of plasticity of microscopically brittle solids (non-associated plastic flow law with two-parameter Mises–Schleicher yield criterion) to describe inelastic strain accumulation in cement stone on the mesoscale. Linear strain hardening was assumed for cement stone (the value of the strain-hardening coefficient is constant). The values of the mechanical characteristics of both components (the elastic constants of basalt and cement stone and the parameters of a unified hardening curve for cement stone) were taken from [73] and are summarized in Table 1 and Table 2, respectively.

Basalt and cement stone are brittle materials. The shear strength of such materials is sensitive to the value of normal/volumetric stress. We use the two-parameter Drucker–Prager fracture criterion to describe local fracture of both components and matrix-aggregate interfaces in the numerical model. The parameters of this criterion are uniaxial compressive strength (UCS) and uniaxial tensile strength (UTS). Table 3 shows the values of these parameters in the simulations. We assume the ideal cohesion of constituents, i.e., strength parameters of matrix-aggregate interfaces are equal to the parameters of the matrix.

### 2.2. Keystones of the Numerical Method

The mechanical behavior of the model concrete samples under loading was modeled using the method of homogeneously (simply) deformable discrete elements [62,74]. The discrete element method (DEM) is based on the representation of a material as an ensemble of interacting (chemically bonded or contacting) particles with a given mass, shape, volume, and surface area [75,76]. Within the framework of the formalism of deformable discrete elements, the last three characteristics can change as a result of element–element interactions.

Each discrete element models some material volume (fragment of a cement stone matrix or basalt aggregate). The dynamics of the simulated sample is determined by the numerical solution of the Newton–Euler system of equations of motion for translational and rotational degrees of freedom of the elements. The authors use the most common approximation in DEM, which applies the equivalent disk/ball approximation to the description of dynamics of a discrete element. This approximation means that the equation of motion of an element is formulated in a simplified form corresponding to a disk (in a two-dimensional formulation of the problem) or a ball (in a 3D case) [75,76,77] (Equation (1)):

(1) $$\{\begin{array}{l} m_{i}\frac{d^{2}{\vec{r}}_{i}}{dt^{2}}=m_{i}\frac{d{\vec{v}}_{i}}{dt}=\sum_{j=1}^{N_{i}}\left({\vec{F}}_{ij}^{n}+{\vec{F}}_{ij}^{\tau }\right)\\ J_{i}\frac{d{\vec{\omega }}_{i}}{dt}=\sum_{j=1}^{N_{i}}{\vec{M}}_{ij} \end{array},$$

where ${\vec{r}}_{i}$, ${\vec{v}}_{i}$, and ${\vec{\omega }}_{i}$ are three-dimensional radius vector, velocity vector, and angular velocity vector of discrete element *i*, *m_i_* is mass of the element, *J_i_* is a moment of inertia of an equivalent disc/ball, ${\vec{F}}_{ij}^{n}$ and ${\vec{F}}_{ij}^{\tau }$ are the potential forces of the central and tangential interaction of the element *i* with the neighboring element *j*, and ${\vec{M}}_{ij}$ is the moment of force. The motion Equation (1) are numerically solved using the velocity Verlet algorithm.

The central and tangential potential forces (${\vec{F}}_{ij}^{n}$ and ${\vec{F}}_{ij}^{\tau }$) are formally written as “independent” although their form is determined on the basis of the constitutive law of the material and is actually consistent. For convenience, the following relationships use specific values of potential interaction forces defined by the ratio of the total values to the area of contact surface (contact area) *S_ij_* (Equation (2)):

(2) $$\{\begin{array}{l} {\vec{\sigma }}_{ij}=\sigma_{ij}{\vec{n}}_{ij}={\vec{F}}_{ij}^{np}/S_{ij}=F_{ij}^{np}{\vec{n}}_{ij}/S_{ij}\\ {\vec{\tau }}_{ij}=\tau_{ij}{\vec{t}}_{ij}={\vec{F}}_{ij}^{\tau p}/S_{ij}=F_{ij}^{\tau p}{\vec{t}}_{ij}/S_{ij} \end{array},$$

where ${\vec{n}}_{ij}$ is unit normal vector oriented along the segment connecting mass centers of the interacting elements *i* and *j* and ${\vec{t}}_{ij}$ is a unit tangential vector in the plane of the contact surface.

The main advantage of the method of homogeneously deformable discrete elements in comparison with the classical formalism of rigid (non-deformable) discrete elements is the fact that they take into account the change (both elastic and irreversible) of the shape and volume of an element under loading. Within the framework of this method, stresses and strains in the volume of an element are assumed to be uniformly distributed and characterized by tensors of averaged stresses and strains. The components of the average stress tensor in the discrete element *i* are calculated as a superposition of the forces of interaction of the element with its neighbors [62,74] (Equation (3)):

(3) $${\bar{\sigma }}_{\alpha \beta }^{i}=\frac{R_{i}}{V_{i}^{0}}\sum_{j=1}^{N_{i}}S_{ij}^{0}\left[\cos\theta_{ij,\alpha }\cos\theta_{ij,\beta }\sigma_{ij}\pm \cos\theta_{ij,\alpha }\sin\theta_{ij,\beta }\tau_{ij}\right],$$

where *R_i_* is the radius of equivalent disk/ball, $S_{ij}^{0}$ is the initial value of the contact area of the element *i* with the neighbor *j* (contact square for unstrained pair), $V_{i}^{0}$ is the volume of the unstrained element *i*, α,β = *x*,*y*,*z* (*XYZ* is laboratory coordinate system), cosθ*_ij_*_,α_ is the projection of the unit normal vector ${\vec{n}}_{ij}$ onto the α-axis, and *N_i_* is the number of neighbors of the element. The components of the average strain tensor ${\bar{\varepsilon }}_{\alpha \beta }^{i}$ in the discrete element *i* can be calculated as a superposition of normal and tangential pair strains by analogy with Equation (3) or incrementally with the use of the material’s constitutive law and calculated average stresses. The latter way is used in the present implementation of the method.

The relations for the forces of element–element interaction are formulated in the general many-body form (Equations (4) and (5)):

(4) $$\{\begin{array}{l} \sigma_{ij}=\sigma_{ij}^{pair}\left(\varepsilon_{jk}\right)+A_{i}{\bar{\sigma }}_{mean}^{i}=\sigma_{ji}=\sigma_{ji}^{pair}\left(\varepsilon_{ji}\right)+A_{j}{\bar{\sigma }}_{mean}^{j}\\ \Delta r_{ij}=\Delta q_{ij}+\Delta q_{ji}=R_{i}\Delta \varepsilon_{ij}+R_{j}\Delta \varepsilon_{ji} \end{array},$$

(5) $$\{\begin{array}{l} \tau_{ij}=\tau_{ij}^{pair}\left(\gamma_{ij}\right)=\tau_{ji}=\tau_{ji}^{pair}\left(\gamma_{ji}\right)\\ \Delta l_{ij}^{sh}=R_{i}\Delta \gamma_{ij}+R_{j}\Delta \gamma_{ji} \end{array}.$$

here, the index “*pair*” denotes pair-wise function, ${\bar{\sigma }}_{mean}^{i}=\left({\bar{\sigma }}_{xx}^{i}+{\bar{\sigma }}_{yy}^{i}+{\bar{\sigma }}_{zz}^{i}\right)/3$ is mean stress in the element *i*, *A_i_* is the material parameter, the symbol Δ denotes an increment of some parameter over the time step of numerical integration of the motion Equation (1), *q_ij_* and *q_ji_* are the distances from the mass centers of the interacting elements *i* and *j* to the central point of the contact area (*q_ij_* = *R_i_* and *q_ji_* = *R_j_* for unstrained elements), ε*_ij_* and ε*_ji_* are central pair strains of discrete elements *i* and *j*, $l_{ij}^{shear}$ is the relative tangential displacement of the elements (it is calculated by taking into account the element rotations), and γ*_ij_* and γ*_ki_* are the shear angles of discrete elements *i* and *j* (contributions of the elements to the total shear angle in the pair *i-j*). Equations (4) and (5) take into account the necessity of satisfying Newton’s third law (σ*_ij_* = σ*_ji_* and τ*_ij_* = τ*_ji_*).

The interaction forces are used in the definition of average stresses and, in turn, are determined by the values of local stresses. This relationship makes possible the implementation of various models of elasticity, plasticity, and fracture (including those with multiparameter criteria) within the framework of the formalism of discrete elements.

### 2.3. Keystones of the Coupled Mechanical Model

The modeled high-strength concrete is a composite material and consists of two components: non-porous basalt aggregates and porous cement stone matrix. Micropores are implemented by removing discrete elements from the generated non-porous model sample (Figure 1).

For modeling fluid-saturated matrix by the method of homogeneously deformable discrete elements, we use a coupled formalism [63]. Within this formalism, a discrete element is considered porous and permeable. Pore space in the discrete element, which models a fragment of cement stone matrix, relates to the capillary porosity of the concrete. The capillary pore space of an element is described implicitly and is characterized by the following integral parameters: pore volume, porosity, and permeability. The fluid in the capillary pore space of an element is taken into account implicitly and is also characterized by integral parameters: mass, density, pore pressure, and dynamic viscosity. The pore fluid is assumed to be Newtonian.

The elastic response of a permeable discrete element modeling a fragment of a cement stone with interstitial fluid is described using Biot’s linear model of poroelasticity [78,79] (Equation (6)):

(6) Δσ¯αβ=2G(Δε¯αβ−δαβaΔPporeK)+δαβ(1−2GK)Δσ¯mean,

where Δ is the increment of the corresponding stress/strain per time step of the numerical scheme for integrating the equations of motion, ${\bar{\sigma }}_{\alpha \beta }$ and ${\bar{\varepsilon }}_{\alpha \beta }$ are averaged stresses and strains in the discrete element, *G* and *K* are the shear and bulk moduli of dry material, δ_αβ_ are Kronecker delta, *P_pore_* is the pressure of fluid in the pore space of the element, and *a* = (1–*K*/*K_s_*) is the poroelasticity coefficient in the Biot’s model (*K_s_* is the bulk modulus of the material of the skeleton walls). Corresponding relationships for the response forces of element *i* to the action of the neighbors *j* are formulated as follows (Equation (7)):

(7) $$\{\begin{array}{l} \Delta \sigma_{ij}=2G_{i}\left(\Delta \varepsilon_{ij}-\frac{a_{i}\Delta P_{pore}^{i}}{K_{i}}\right)+\left(1-\frac{2G_{i}}{K_{i}}\right)\Delta {\bar{\sigma }}_{mean}^{i}\\ \Delta \tau_{ij}=2G_{i}\Delta \gamma_{ij} \end{array},$$

where *G_i_*, *K_i_*, *a_i_*, and *P^i^_pore_* are corresponding parameters for the element *i*.

The pore fluid is assumed to be linearly compressible. The main constitutive equations relating capillary pore pressure in the element to average mean stress, pore volume, and fluid density within the framework of Biot’s model, are as follows [78,79] (Equations (8) and (9)):

(8) $$\frac{V_{p}-V_{p}^{0}}{V}=\varphi -\varphi_{0}=\frac{a}{K}\left({\bar{\sigma }}_{mean}-{\bar{\sigma }}_{mean}^{0}\right)+\left(\frac{1}{K}-\frac{1+\phi }{K_{s}}\right)\left(P_{pore}-P_{pore}^{0}\right),$$

(9) $$P_{pore}=P_{pore}^{0}+K_{fl}\left(\frac{\rho_{fl}}{\rho_{fl}^{o}}-1\right)=P_{pore}^{0}+K_{fl}\left(\frac{m_{fl}}{\rho_{fl}^{o}V_{p}}-1\right).$$

here, *V_p_* and $V_{p}^{0}$ are the current and initial values of the pore volume in the element, φ and φ_0_ are the corresponding values of porosity, *V* is the volume of the element, $\rho_{fl}^{0}$ and $P_{pore}^{0}$ are the equilibrium values of the density and pressure of the fluid under atmospheric conditions, *m_fl_* and ρ*_fl_* are the current values of fluid mass and density in the capillary pore space of the element, and *K_fl_* is the bulk modulus of fluid.

The inelastic behavior of mesoscale volumes of cement stone under loading is mainly due to the occurrence, accumulation, and growth of micro- and nanoscale damage. We applied the non-associated plastic flow law with the Mises–Schleicher yield criterion (Nikolaevsky’s model) [62,63,80] to describe stress relaxation and accumulation of inelastic strains in the cement stone. This model is widely used for natural and artificial brittle materials [81,82]. The effect of pore pressure in the volume of a discrete element that simulates a cement stone on its inelastic mechanical behavior is taken into account using a modified formulation of the Mises–Schleicher yield criterion (Equation (10)):

(10) $$\Phi =\alpha \left({\bar{\sigma }}_{mean}+P_{pore}\right)+{\bar{\sigma }}_{eq}/\sqrt{3}=Y,$$

where α is the internal friction coefficient, *Y* is cohesion (yield stress under simple shear loading), and ${\bar{\sigma }}_{eq}=\frac{1}{\sqrt{2}}\sqrt{{\left({\bar{\sigma }}_{xx}-{\bar{\sigma }}_{yy}\right)}^{2}+{\left({\bar{\sigma }}_{yy}-{\bar{\sigma }}_{zz}\right)}^{2}+{\left({\bar{\sigma }}_{zz}-{\bar{\sigma }}_{xx}\right)}^{2}+6\left[{\left({\bar{\sigma }}_{xy}\right)}^{2}+{\left({\bar{\sigma }}_{yz}\right)}^{2}+{\left({\bar{\sigma }}_{xz}\right)}^{2}\right]}$ is the equivalent (Mises) stress. The feature of Nikolaevsky’s model is a postulated linear dependence of plastic volume strain rate. The dimensionless coefficient of proportionality Λ is called the coefficient (rate) of dilatancy.

Nikolaevsky’s model is implemented within the explicit numerical scheme of solving motions Equation (1) using the well-known radial return algorithm of Wilkins [83]. The essence of this algorithm is the scaling of the components of the deviator stress tensor at each integration step after finishing the elastic problem solution. Being written in terms of stress, the scaling expression for the applied model of plasticity has the following form (Equations (11) and (12)):

(11) $${{\bar{\sigma }}^{\prime }}_{\alpha \beta }=\left({\bar{\sigma }}_{\alpha \beta }-\delta_{\alpha \beta }{\bar{\sigma }}_{mean}\right)M+\delta_{\alpha \beta }{{\bar{\sigma }}^{\prime }}_{mean},$$

(12) $$\{\begin{array}{l} {{\bar{\sigma }}^{\prime }}_{eq}={\bar{\sigma }}_{eq}-\frac{\sqrt{3}GC}{K\Lambda \alpha /3+G}\\ {{\bar{\sigma }}^{\prime }}_{mean}={\bar{\sigma }}_{mean}-\frac{1}{3}\frac{K\Lambda C}{K\Lambda \alpha /3+G} \end{array},$$

where C=Φ−Y, $M=\sqrt{{{\bar{\sigma }}^{\prime }}_{eq}/{\bar{\sigma }}_{eq}}$, ${{\bar{\sigma }}^{\prime }}_{mean}$ and ${{\bar{\sigma }}^{\prime }}_{eq}$ are “relaxed” (scaled) values of mean stress and equivalent stress in the element. To provide the required scaling of average stresses in the element *i*, all central and tangential forces of the response of the element *i* to the impact of the neighbors *j* are scaled using a ratio similar to Equation (11) (Equations (13) and (14)):

(13) $$\{\begin{array}{l} {\sigma^{\prime }}_{ij}=\left(\sigma_{ij}^{cur}-{\bar{\sigma }}_{mean}^{i}\right)M_{i}+({\bar{\sigma }}_{mean}^{i}-L_{i})\\ {\tau^{\prime }}_{ij}=\tau_{ij}^{cur}M_{i} \end{array},$$

(14) $$\{\begin{array}{l} M_{i}=1-\frac{\sqrt{3}}{{\bar{\sigma }}_{eq}^{i}}\frac{G_{i}C_{i}}{K_{i}\Lambda_{i}\alpha_{i}/3+G_{i}}\\ L_{i}=\frac{1}{3}\frac{K_{i}\Lambda_{i}C_{i}}{K_{i}\Lambda_{i}\alpha_{i}/3+G} \end{array},$$

where $\left({\sigma^{\prime }}_{ij},{\tau^{\prime }}_{ij}\right)$ are scaled specific central and tangential (shear) response forces of the element *i*.

The unified hardening curve for the cement stone is defined by the dependence $Y\left(\varepsilon_{ms}\right)$, where $\varepsilon_{ms}=\left(K\varepsilon_{mean}+P_{pore}\right)G\alpha /3+\varepsilon_{eq}/\sqrt{3}$ is a “Mises–Schleicher strain parameter”. The form of parameter $\varepsilon_{ms}$ ensures the equality $\left(\alpha \left({\bar{\sigma }}_{mean}+P_{pore}\right)+{\bar{\sigma }}_{eq}/\sqrt{3}\right)/\varepsilon_{ms}=3G$ within the region of elastic strain similar to the conventional model of plasticity of metals with von Mises yield criterion. The strain-hardening coefficient is the derivative $dY\left(\varepsilon_{ms}\right)/d\varepsilon_{ms}$. More details of numerical implementation of the above mechanical model of inelastic behavior of brittle solids can be found in [62,63].

The discrete element model of a concrete sample in an initial (unstrained) state assumes that all neighboring elements are chemically bonded (no mesoscale cracks). Local fracture under loading was modeled by breaking the chemical bond between the elements when the specified fracture criterion is satisfied [62,63]. We applied the two-parameter Drucker–Prager criterion [84] for cement stone (this classical criterion was also modified by taking into account the local pore pressure) (Equation (15)):

(15) $$\sigma_{fract}=1.5\left(\beta -1\right)\left(\sigma_{mean}+P_{pore}\right)+0.5\left(\beta +1\right)\sigma_{eq}=\sigma_{c},$$

where *β* = σ*_c_*/σ*_t_*, σ*_c_*, and σ*_t_* are the values of UCS and UTS for dry materials. Numerical implementation of this criterion in the discrete element method is based on determining the local stress tensor on the contact area of the linked pair of elements and calculating its invariants [62]. These invariants are calculated for the pair at each time step and then substituted into Equation (15) to check the bond break condition is met in the pair.

Basalt is assumed to be a non-porous, elastic-brittle material. Its mechanical behavior was described using the generalized Hooke’s law Equations (6) and (7) and the Drucker–Prager fracture criterion Equation (15) at *P_pore_* = 0. Fracture of interfaces (the pairs of elements with one element modeling cement stone and the other modeling aggregate) was considered using the Drucker–Prager criterion with the material parameters σ*_c_* and σ*_t_* corresponding to cement stone.

The pore pressure gradient was considered the driving force of liquid redistribution in the capillary pore space of the cement stone. Filtration was described by numerically solving the equation of fluid transport [85]. We used the form of the equation that takes into account the finite velocity of propagation of disturbances (Equation (16)):

(16) $$\vec{\upsilon }=-\frac{k}{\eta }\nabla P_{pore}-\tau_{r}\frac{\partial \vec{\upsilon }}{\partial t}\;\mathrm{where}\;\tau_{r}=\frac{K_{fl}}{\phi }\frac{k}{\eta }\frac{1}{V_{fl}^{2}}.$$

here, $\vec{\upsilon }$ is the power density of the fluid flow, *k* is the capillary permeability of the element, η is the fluid viscosity, τ*_r_* is the relaxation time, and $V_{fl}=\sqrt{K_{fl}/\rho_{fl}^{0}}$ is the sound velocity in the fluid. Equation (6) was numerically solved by the finite volume method on an ensemble of discrete elements [63].

Fluid transport between the micropore and surrounding discrete elements of the cement stone (the walls of the micropore) is calculated similarly to the calculation of the transport of pore fluid between adjacent discrete elements, namely with the use of Equation (16). In the numerical solution of Equation (16) for the pairs “micropore–surface discrete elements”, the micropore is replaced by a virtual discrete element with porosity 1, permeability 4R^2^ (*R* is discrete element radius), and pore pressure equal to the real pressure of fluid in the micropore. The outflow of fluid from the sample into the surrounding space (through external surfaces) or into new internal voids resulting from the formation of damages/cracks and damage/crack surfaces separation is calculated in a similar way.

### 2.4. Loading Conditions and Varied Material Parameters

We numerically studied the effect of free pore water on the value of dynamic strength and fracture of 2D mesoscale samples of high-strength concrete under uniaxial compression. 2D concrete samples were modeled in the plane stress approximation (σ*_zz_* = σ*_xz_* = σ*_yz_* = 0, where *XY* is the plane of deformation). In some respects (in particular, with respect to the stiffness of the sample), this corresponds to the conditions of a real experiment on concrete cubic samples.

2D concrete samples were modeled as an ensemble of close-packed elements of the same size. The size of the discrete element was 500 μm and is equal to the size of the micropore (each micropore was created by removing one discrete element from the matrix). The samples were of square form. The “basic” value of the sample size (side length) was 10 cm.

**Figure 2:**
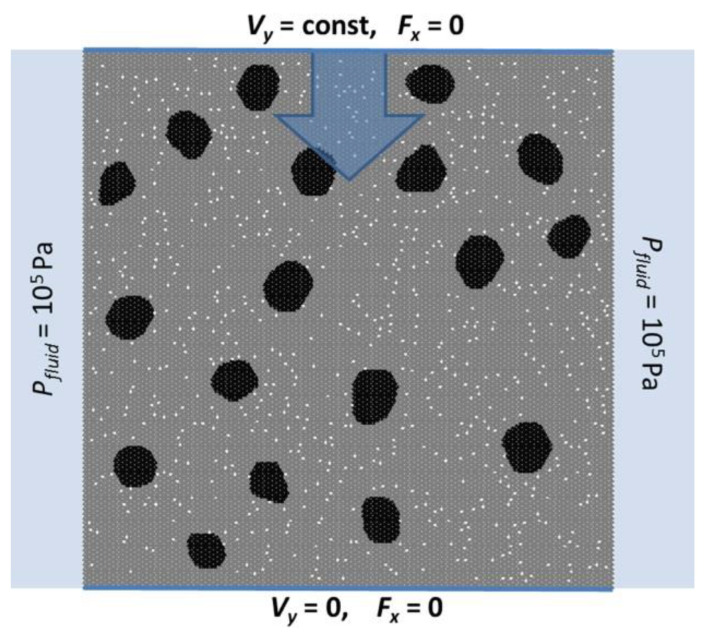
Schematic of uniaxial compression of concrete sample.

We modeled uniaxial compression of the samples at a constant loading velocity *V* (Figure 2). The lower (support) surface of the sample was fixed in the vertical direction and the upper surface was displaced in the vertical direction at a velocity *V_y_*. Zero external forces were applied to both (the upper and lower) surfaces in the horizontal direction to fulfill the boundary condition σ*_xx_* = σ*_xy_* = 0.

Side surfaces of the sample were stress-free and hydraulically open to provide the possibility of a free outflow of fluid from the sample.

The initial (before loading) values of the pore fluid density and pressure in all capillary pores and micropores were equal to 1000 kg/m^3^ and atmospheric pressure, respectively. The space surrounding the sample was filled with the same fluid at atmospheric pressure, which was supported constantly throughout the loading.

To vary the balance of the processes of deformation of the pore space of concrete and pore fluid flow, we modeled uniaxial compression at different values of the loading velocity *V_y_*, the permeability *k* of the capillary system in the cement stone (it characterizes the leak-off capacity of concrete), the size of the sample *W*, and the dynamic viscosity of the pore fluid η (which can potentially be fresh or saltwater, as well as an aqueous solution). We varied the strain rate $\dot{\varepsilon }=V_{y}/W$ within three orders of magnitude (from 1·10^−3^ s^−1^ to 5 s^−1^), the viscosity of the fluid η within an order of magnitude relative to the viscosity of saline water (2·10^−4^ Pa·s to 3·10^−3^ Pa·s), the permeability *k* within two orders of magnitude (from 6·10^−16^ m^2^ to 6·10^−14^ m^2^), and the sample size *W* within four times (from 5 cm to 20 cm).

We measured the compressive strength of a sample as the peak value of sample resistance force to the upper surface divided by the end surface area. The fracture pattern was analyzed qualitatively and quantitatively using the information about the spatial distribution and the number of broken bonds in the matrix, aggregates, and interfaces.

## 3. Results

In this work, we consider the range of strain $\dot{\varepsilon }$ rates in which the mechanical characteristics of dry concrete samples (including strength) can be reasonably assumed to be strain rate independent. This particularly determined the choice of rate-independent formulation of strength criterion Equation (5). At the same time, mobile fluid in the pore space is capable of changing the value of critical (maximum) load depending on the loading rate. The expanding action of the pore fluid causes an earlier (in comparison with “dry” samples) onset of damage initiation and the development of cracks in the material, and thus determines a reduced strength value. During the process of material compression, the pore space and the pore fluid are both compressed. This leads to an increase in pore pressure (and its gradient), which not only affects the stress state of the skeleton but initiates fluid flow. The outflow of pore fluid through free side surfaces (due to a gradually growing pressure gradient from the center of the sample to the periphery during compression) promotes a decrease in pore pressure. During the course of material compression, there is competition between two interrelated and oppositely directed processes that control the dynamics of pore pressure growth. Due to the inertia of the fluid flow process, it is reasonable to suppose that the value of the dynamic strength of porous fluid-saturated concrete samples should decrease with an increase in the strain rate. In the quasi-static regime of loading (fully drained condition), the strength of concrete should be close to the upper limit corresponding to the strength of the dry sample [21]. As the compression rate increases, the strength value should tend to the lower limit corresponding to the strength of water-saturated hydro-isolated samples (undrained condition) [24,63].

The competitive influence of compression of the porous skeleton (this leads to an increase in pore pressure) and filtration of pore fluid (this leads to a decrease in pore pressure) can be qualitatively and quantitatively characterized by the ratio of the time scales of these processes. The time scale of the first process can be characterized by the inverse strain rate: $T_{def}\sim 1/\dot{\varepsilon }$. The time scale of the fluid flow process (called the Darcy timescale) is defined as $T_{Darcy}=\eta W^{2}/k\left(P_{2}-P_{1}\right)$, where *W* is a characteristic system lengthscale, (*P*_2_ − *P*_1_) is pore pressure difference at the ends of this lengthscale (pressure gradient is driving force for fluid flow), *η* is fluid viscosity, and *k* is the material permeability. Note that the given relation for Darcy’s time scale is derived by the finite-difference approximation of Darcy’s law. The ratio of these times (dimensionless Darcy number [24,25]) characterizes the balance of the rates of deformation and filtration (Equation (17)):

(17) $$Da=\frac{T_{Darcy}}{T_{def}}=\frac{\eta \dot{\varepsilon }W^{2}}{k\left(P_{2}-P_{1}\right)},$$

We were the first to show that the dimensionless parameter *Da* can be considered a generalization of the conventional dynamic parameter $\dot{\varepsilon }$ for fluid-saturated high-strength concretes with two-scale pore structure. In a recent paper [65], we showed that the dependence of dynamic compressive strength of high-strength concrete σ*_comp_* on strain rate has a nonlinear (logistic) profile. We discussed the advantage of using the generalized dependence σ*_comp_*(*Da*) instead of conventionally used σ*_comp_*($\dot{\varepsilon }$). The dependence σ*_comp_*(*Da*) takes into account the scale factor through the size *W* and is applicable for various pore fluids and concrete permeability values (at a constant porosity).

The present study is focused on revealing and analyzing the relation of the features of this generalized strength dependence to the features and mechanisms of fracture of fluid-saturated concrete. The analysis is based on the simulation results for various values of $\dot{\varepsilon }$, η, *k*, and *W* (the limits of their variation are given above).

Concrete is a heterogeneous composite material with two-scale porosity and mesoscale hardening inclusions. These structural factors should have a significant influence on the value of the dynamic strength and the limits of its change. To identify the contribution of each of them, the following were analyzed:

1. The role and contributions of different scale pore subsystems (micropores and capillary network). The analysis of this factor was carried out on the basis of the results of modeling samples without micropores (only a capillary network) and with micropores.
2. Inhomogeneity of the structure at the mesoscale. Samples with the different spatial distribution of micropores and basalt aggregates were analyzed, and the intervals of scatter of local dynamic strength (strength of mesoscopic samples) were estimated.

### 3.1. Generalized Rate Dependence of Strength for Concretes with One-Scale and Two-Scale Porosity

The considered high-strength concrete has two-scale porosity but the characteristic fluid flow rate is mainly determined by the permeability of the capillary pore network *k*. To study the effect of cement stone permeability (provided by a connected network of capillary pore channels) on the compressive strength of concrete, we first simulated uniaxial compression of model samples with cement stone containing only capillary porosity (no micropores). The typical initial structure of such samples corresponds to that presented in Figure 1 but without micropores.

The results of modeling the mesoscale samples of fluid-saturated concrete with one-scale (capillary) porosity at various values of $\dot{\varepsilon }$, η, *k*, and *W* confirm the correctness of the assumption [65] that the dynamic compressive strength is a single-valued function of the ratio of the characteristic rates of deformation and fluid flow (*Da*). Note that the difference (*P*_2_–*P*_1_) in (17) has the meaning of the pressure difference in the center of the sample and on the free side surface in the direction transverse to the loading axis. When applying the parameter *Da* to uniquely characterize dynamic strength, we should take into account that the pore pressure in the center of the sample (*P*_2_) continuously increases during the course of sample compression, while it remains constant and equal to atmospheric pressure (*P*_1_ = 10^5^ Pa) on the side surface. Hence, the characteristic value of fluid flow rate continuously increases due to a gradual increase in the pressure difference in the sample. We therefore use an analog of the Darcy number Equation (17) with *P*_2_ calculated as Terzaghi’s estimation of maximum reachable pore pressure: $P_{2}=\sigma_{c}^{Cem}\phi_{0}$, where φ_0_ is the initial capillary porosity of the cement stone (*P*_2_~7 MPa for simulated cement stone). The pressure *P*_1_ in Equation (17) is pore pressure on the side surface of the sample (*P*_1_ = 10^5^ Pa).

**Figure 3:**
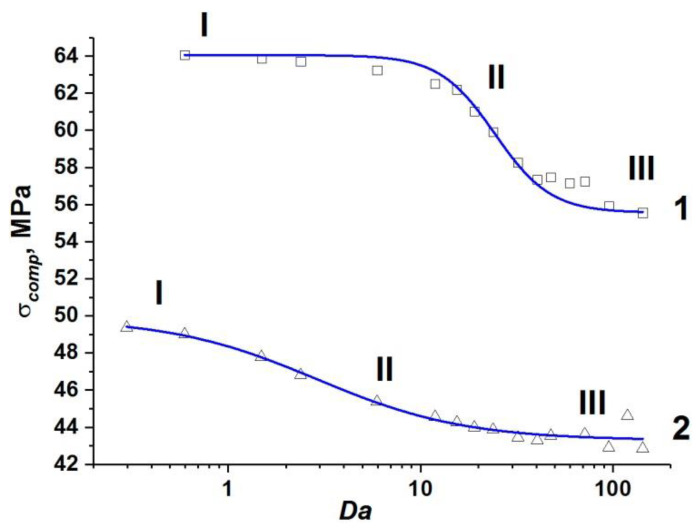
Dependence of the compressive strength of fluid-saturated mesoscale concrete samples with one-scale (curve 1) and two-scale (curve 2) pore structures on the Darcy number *Da*. Three main sections are marked on the curves: the characteristic fluid flow times are much shorter than the characteristic loading times (I); a qualitative change in the balance of these competing processes (II); the characteristic fluid flow times are much longer than the characteristic loading times (III).

The set of obtained values of the compressive strength of concrete samples with one-scale (capillary) porosity at different values of $\dot{\varepsilon }$, η, *k*, and *W* form a unified curve σ*_comp_*(*Da*) (curve 1 in Figure 3). This curve has a logistic form and is well approximated by a sigmoid function (Equation (18)):

(18) $$\sigma_{comp}\left(Da\right)=\sigma_{comp}^{\min}+\frac{\sigma_{comp}^{\max}-\sigma_{comp}^{\min}}{\left(1+{\left[Da/24.37\right]}^{3}\right)},$$

where σ*_comp_* is the dynamic compressive strength of the concrete sample; $\sigma_{comp}^{\min}$ ≈ 55.6 MPa is the minimum value of the strength of the concrete sample, namely, at negligible filtration (at high loading rates or low permeability); $\sigma_{comp}^{\max}$ ≈ 64 MPa is the maximum value of the strength equal to the strength of a dry sample or a water-saturated sample under quasi-static loading (when the outflow of pore fluid through the side surfaces maintains a pore pressure equal to atmospheric). The characteristic range of change in the strength value is ($\sigma_{comp}^{\max}-\sigma_{comp}^{\min}$) ≈ 8 MPa, which is about 15% of the compressive strength of dry concrete. We should remind ourselves here that the strength of the dry sample can be reasonably assumed to be constant ($\sigma_{comp}^{\max}$ ≈ 64 MPa) in the considered range of strain rates.

The unified dependence σ*_comp_*(*Da*) (curve 1 in Figure 3) can be divided into three characteristic sections.

Section I (*Da* < 10) corresponds to such combinations of values of structural parameters, fluid viscosity, and loading rate, which ensure enough time for fluid redistribution in the volume of capillary pore channels and flow out of the sample. Therefore, the pore pressure is at a low level. In this case, the filtration (outflow) of the fluid is an efficient mechanism of stress relaxation in the skeleton, and the strength of the sample is close to the maximum value and changes only slightly. The maximum strength (~64 MPa) is achieved at *Da*→ 0.

Section III (*Da >* 100) corresponds to the combinations of parameters $\dot{\varepsilon }$, η, *k*, and *W*, at which the fluid does not have time to redistribute in the capillary network during concrete loading. The sample is actually under undrained conditions, and the pore pressure is close to the maximum achievable value, thus minimizing the strength of concrete as much as possible. Therefore, the concrete strength remains almost unchanged at *Da >* 10 and is close to the minimum value (~56 MPa).

Section II of the curve (10 < *Da <* 100) is transitional, the ratio of strain rate to the rate of pore fluid filtration varies within the above-mentioned limits.

**Figure 4:**
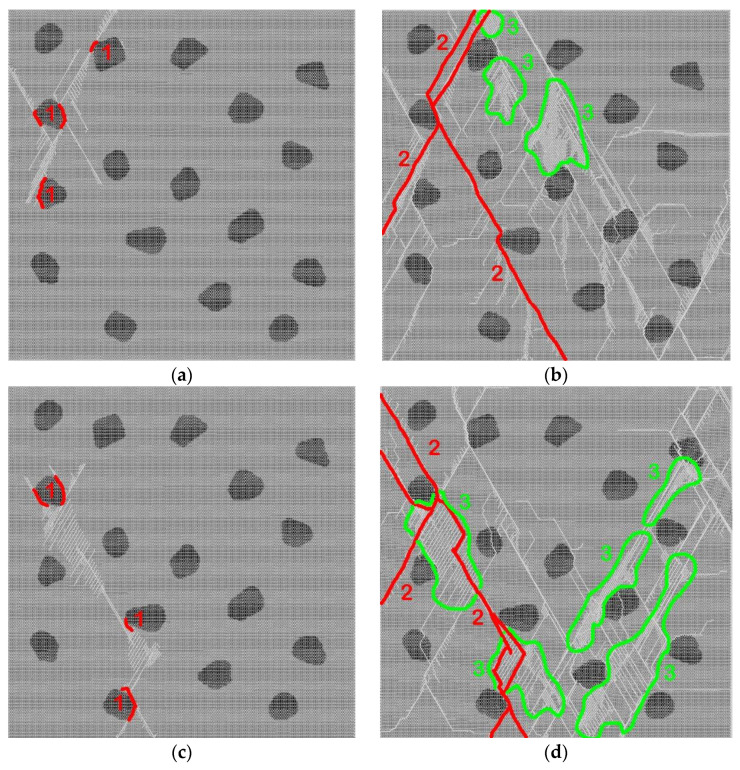
Fracture of model water-saturated concrete samples with capillary pores at different values of *Da*: 0.5 (**a**,**b**); 140 (**c**,**d**). Figuers (**a**,**c**) show the beginning phase of fracture, while figures (**b,d**) show broken samples. Primary (interfacial) damages in (**a**,**c**) are marked by the red lines and indicated by the number 1. The main cracks (first destructive cracks) in (**b**,**d**) are marked by the red line and indicated by the number 2. The cleavage zones are bordered by green contours and indicated by the number 3.

Analysis of fracture of samples characterized by different values of the Darcy number indicates their qualitatively similar patterns corresponding to typical fracture of brittle heterogeneous materials (Figure 4). The first damages nucleate and accumulate mainly at the interfaces between the “soft” matrix and “hard” aggregates (Figure 4a), where the maximum deviator stresses are localized. These interface damages and small cracks inhibit the load transfer from the matrix to the inclusions. This leads to the development of fracture through the growth of cracks in the cement stone. The sources of these cracks are the above-mentioned interface damages. Since the characteristic value of the inelastic strain of the cement stone is relatively small, the dynamics of matrix fracture is close to that of brittle materials. It is characterized by the fast propagation of main cracks and by the formation of many secondary (local) cracks (Figure 4a,b). We emphasize the formation of small zones of strong fragmentation (cleavage up to crushing) of the matrix. The formation of cleavage zones is an effective way to relax the high local shear stresses in the regions constrained by intersecting inclined long cracks.

It is important to note the changes in the fracture pattern with increasing loading rate characterized by the Darcy number (Figure 4c,d). An increase in the Darcy number (and a corresponding decrease in the role of pore fluid flow and increase in the rate of pore pressure growth) is accompanied by an increase in the “multiplicity” of fracture. The number of main cracks and connecting secondary (local) cracks increases with the value of *Da*. The number and characteristic size of cleavage zones in the cement stone also increase significantly. Such a change in the fracture pattern (an increase in the degree of fragmentation with an increase in the generalized loading rate parameter *Da*) qualitatively corresponds to the experimentally observed regularities for various water-saturated brittle materials including concretes [10,31,35,36,86].

To study the contribution of micropores to the strength of concrete, we considered mesoscopic samples with two-scale porosity of cement stone (Figure 1). As noted above, the micropore diameter was 500 μm and the microporosity value was 4% of the total volume of the cement stone. Micropores and capillary pores were assumed to be connected (combined into a single filtration system).

Analysis of the simulation results at various values of $\dot{\varepsilon }$, η, *k*, and *W* shows that the strength of water-saturated concrete with two-scale porosity is also a single-valued function of *Da*. Similar to the case of one-scale porosity, this dependence is well approximated by a sigmoid function (Equation (19)):

(19) $$\sigma_{comp}=\sigma_{comp}^{\min}+\frac{\sigma_{comp}^{\max}-\sigma_{comp}^{\min}}{\left(1+{\left[Da/2.90\right]}^{1.8}\right)},$$

where $\sigma_{comp}^{\min}$ ≈ 43.3 MPa and $\sigma_{comp}^{\max}$ ≈ 49.9 MPa (curve 2 in Figure 3).

One can see that the effect of microporosity on the change in compressive strength of concrete is manifested in the entire considered range of variation of the parameter *Da*.

First of all, it is important to mention a significant overall decrease in the value of dynamic compressive strength of the samples. The maximum and minimum strength values of fluid-saturated concrete with two-scale porosity ($\sigma_{comp}^{\max}$ and $\sigma_{comp}^{\min}$) decrease by approximately the same amount (13–14 MPa or about 25%) in comparison with similar strength values for concrete with only capillary pores in the mortar. An explanation of this effect is clear. Micropores in the concrete structure are mesoscopic stress concentrators and cause earlier initiation of damages and cracks in the cement stone and the formation of the main crack at lower values of the applied load.

At the same time, the dynamics of change in the sample strength from $\sigma_{comp}^{\max}$ to $\sigma_{comp}^{\min}$ significantly differs from the same for concretes with only capillary pores (Figure 3). The key difference is that the length of section I contracts to 0 < *Da* < 0.4. However, stage III (minimum strength values) begins at the same characteristic values of the Darcy number as in the case of concrete with a one-scale pore system (*Da* > 100). In other words, large-scale pores (micropores) provide an increase in the length of the transition stage II by almost two orders of magnitude.

**Figure 5:**
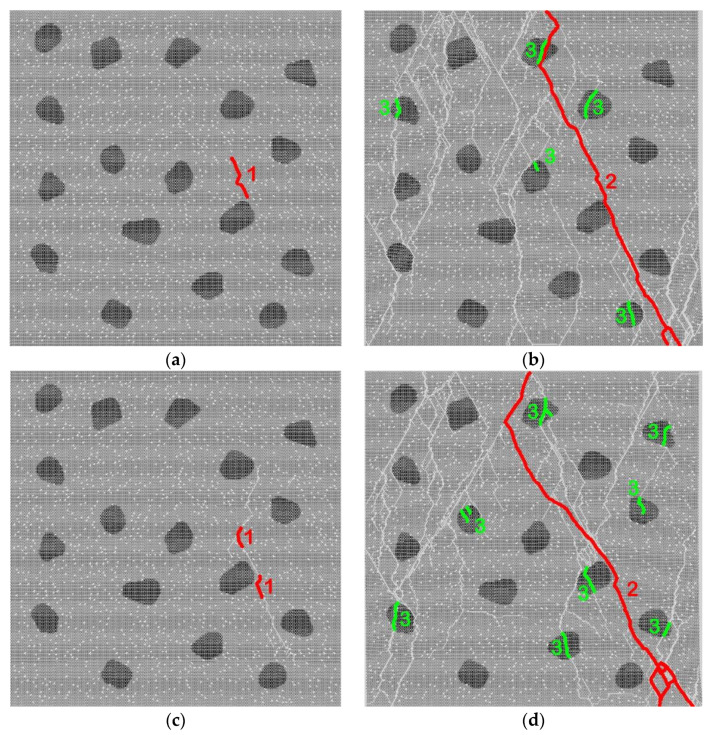
Fracture of model water-saturated concrete samples with capillary and microscopic pores at different values of *Da*: 0.5 (**a**,**b**); 140 (**c**,**d**). Figures (**a**,**c**) show the beginning phase of fracture, while figures (**b**,**d**) show broken samples. Primary short cracks originating from damage to the mesopore walls are shown in (**a**,**c**). They are marked by the red lines and indicated by the number 1. The main cracks in (**b**,**d**) are marked by the red line and indicated by the number 2, the intraparticle cracks (green lines) are indicated by the number 3.

Fracture of concrete samples with two-scale porosity (Figure 5) is much more localized in comparison with concrete with one-scale porosity (Figure 4).

All fractures in the matrix, including the first damage (Figure 5a,c), destroying main cracks, and connecting internal cracks (Figure 5b,d), are related to micropores. Primary damages are formed by the destruction of the walls of micropores. Under the increasing load, adjacent damage coalesces into short internal cracks (Figure 5a,c) and then into long cracks up to main cracks (Figure 5b,d). Note that the growth of cracks is discrete and occurs in a jump-like manner (from micropore to micropore). Two important differences can be distinguished between the fracture pattern of specimens with two-scale porosity and the fracture pattern of specimens with only capillary porosity. First, when meeting a hard inclusion of basalt, a growing crack often “cuts” it but does not bend around it (Figure 5b,d). Second, we emphasize a qualitatively higher degree of fracture localization including a smaller number of short secondary cracks connecting long cracks, and the absence of cleavage zones. This is a consequence of the large number of centers of damage nucleation (micropores), which allow more efficient relaxation of high local shear stresses by localizing fracture (crack formation).

We also emphasize the systematic change in aggregate contribution to fracture with an increase in *Da*. Fracture of aggregates is predominantly at the interfaces (interfacial cracks, which partially detach inclusions from the matrix) at small *Da* values, although the number of intraparticle damages, short or through cracks (cracks that pass through the inclusion) is larger than in concrete with only capillary porosity. An increase in *Da* and a corresponding increase in characteristic pore pressure in the matrix are accompanied by a nearly twofold increase in the number of through intraparticle cracks and a concurrent decrease in the number of small damages in the particles. The number of intraparticle cracks at large *Da* values is comparable to the number of cracks that propagate around the particles through the interface.

### 3.2. Influence of Heterogeneity of Concrete Structure on the Value of Local (Mesoscopic) Dynamic Compressive Strength

The previous section analyzes the general features of the mechanical effect of pore fluid pressure and pore structure parameters on the dynamic compressive strength of a mesoscopic representative volume of high-strength concrete. Concrete is a strongly heterogeneous material. The heterogeneity of the structure can differ significantly in different mesoscale regions of large-scale (macroscopic) structures due to the features of manufacturing technology. Direct averaging of the strength values of various statistically equivalent mesoscale volumes does not guarantee an accurate estimate of the macroscopic strength of concrete, since macroscopic strength is determined, among other things, by the interaction of adjacent mesovolumes. At the same time, knowledge of the local (mesoscopic) characteristics of concrete is of most importance in many topical practical problems. Examples of such problems are local contact loading of underwater parts of large concrete structures, including the collision of a ship with a concrete berth or dynamic contact load by ice plates during “icequakes” (analog of earthquakes in ice covers of large northern lakes or seas) [38,39,87]. Underestimation of local dynamic strength can have the far-reaching consequence of reducing the operational life of the entire concrete structure.

The foregoing determines the relevance of estimating the characteristic interval of variation of the local dynamic strength of statistically equivalent mesoscale concrete volumes characterized by the different spatial distributions of structural elements. Variation in the mechanical properties of concrete at the mesoscale is determined by the different spatial arrangements of micropores in cement stone and hardening aggregates. To identify the partial contribution of each of these factors to the dynamic strength of concrete, we carried out two series of calculations:

3 The first series investigated the strain rate dependence of the dynamic strength for five model samples with different spatial positions of micropores. Note that the combined effect of micropores and basalt aggregates on the strength of concrete is generally not a superposition of their partial contributions. Therefore, we analyzed the influence of the spatial arrangement of micropores on the variation in the strength of cement stone (the main component of concrete).
4 The second series investigated the strain rate dependence of the dynamic strength for five model concrete specimens with different spatial arrangements of aggregates. All concrete samples were characterized by the same spatial position of micropores in the cement stone (except for the areas occupied by basalt aggregates).

Other mechanical and geometric parameters of the samples, as well as the loading conditions, are the same as those used in the previous section.

**Figure 6:**
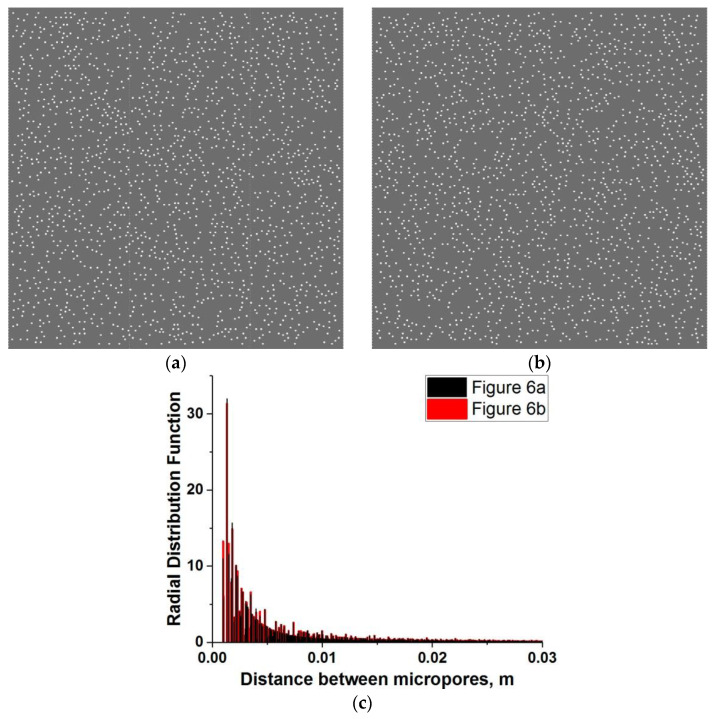
Examples of the initial structure of fluid-saturated microporous (4 vol.%) samples of cement stone with different spatial arrangements of micropores (**a**,**b**) and corresponding radial distribution functions of micropores (**c**).

Figure 6a,b show examples of model cement stone specimens. Different spatial positions of micropores in the samples were provided by setting different seed values of the random number generator in the micropore generating software. The statistical equivalence of the pore distributions in these samples is confirmed by the coincidence of the radial distribution functions (Figure 6c).

The results of modeling uniaxial compression at various values of $\dot{\varepsilon }$, η, *k*, and *W* shows that strain rate depends on the compressive strength of the representative mesovolumes with different spatial positions of micropores are well approximated by sigmoid functions. The approximating functions for different cement stone samples are almost parallel to each other, and their parameters (minimum and maximum strength, exponent, and position of the midpoint) differ insignificantly. The sigmoids below correspond to the “limiting” cases (upper and lower boundary curves) (Equations (20) and (21)):

(20) $$\sigma_{comp}=\sigma_{comp}^{\min}+\frac{\sigma_{comp}^{\max}-\sigma_{comp}^{\min}}{\left(1+{\left[Da/6.31\right]}^{1.9}\right)},$$

where $\sigma_{comp}^{\min}$ ≈ 50.41 MPa, $\sigma_{comp}^{\max}$ ≈ 56.86 MPa;

(21) $$\sigma_{comp}=\sigma_{comp}^{\min}+\frac{\sigma_{comp}^{\max}-\sigma_{comp}^{\min}}{\left(1+{\left[Da/4.51\right]}^{1.85}\right)},$$

where $\sigma_{comp}^{\min}$ ≈ 47.85 MPa, $\sigma_{comp}^{\max}$ ≈ 54.13 MPa.

**Figure 7:**
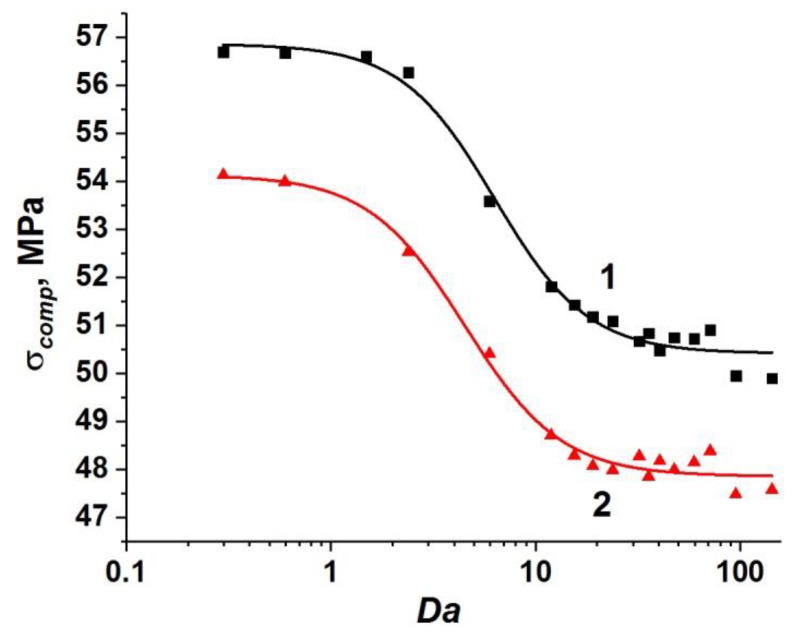
Examples of the dependence of compressive strength of water-saturated cement stone samples with different spatial arrangements of micropores (4 vol.%). Curves 1 and 2 correspond to the samples in Figure 6a,b, respectively.

Equations (20) and (21) are shown in Figure 7. They correspond to the samples in Figure 6a,b, respectively. Curves σ*_comp_*(*Da*) for the rest of the samples lie within the interval limited by these two “limiting” curves. One can see that the range of variation in the value of the local dynamic strength of the cement stone is nearly constant in the entire range of *Da* and is ~5%.

**Figure 8:**
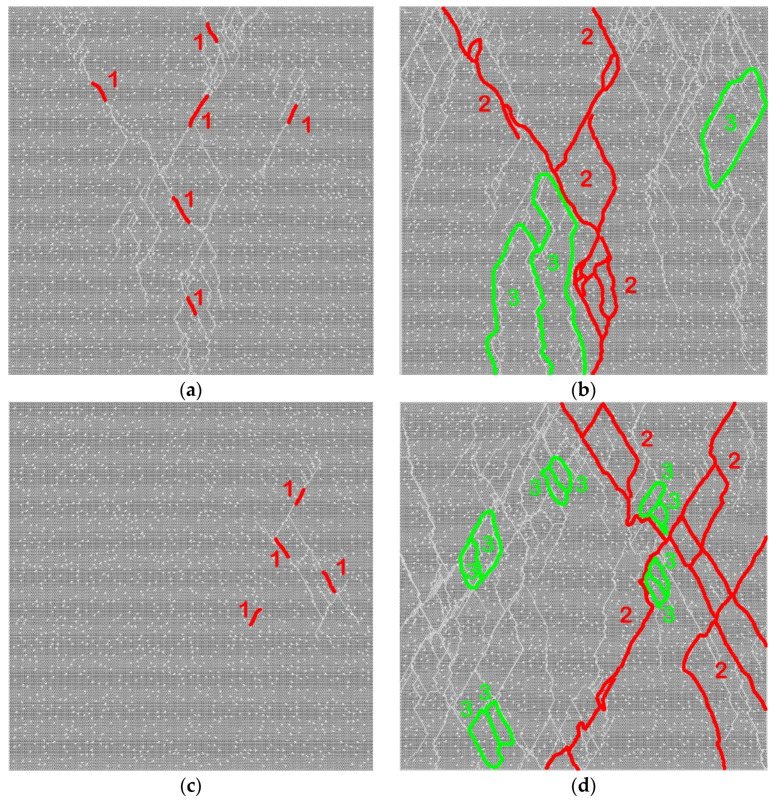
Fracture of model water-saturated samples of cement stone with a two-scale pore structure at different values of *Da*: 0.5 (**a**,**b**); 140 (**c**,**d**). Figures (**a**,**c**) show the beginning phase of fracture, while figures (**b**,**d**) show broken samples. Primary short cracks originating from damage to the mesopore walls are shown in (**a**,**c**). They are marked by the red lines and indicated by the number 1. The main cracks in (**b**,**d**) are marked by the red line and indicated by the number 2. Some typical non-equiaxial fragments are bordered with green contours and indicated by the number 3.

Samples of cement stone with different spatial arrangements of micropores have a similar pattern of fracture at the same values of *Da*, and the change in fracture pattern is determined by the value of *Da*. At all *Da* values, the first damages appear on the walls of micropores (Figure 8a,c) because the micropore is the softest mesoscopic structural element. With a further increase in the load, these damages coalesce into small cracks that develop into main cracks and numerous secondary cracks that provide material fragmentation (Figure 8b,d). Note that cracks develop in a jump-like manner (from micropore to micropore). They have complex trajectories and break the sample into non-equiaxial blocks oriented along the loading direction. Secondary (connecting) cracks divide large blocks into smaller fragments. Note that the development of a system of secondary cracks is much more pronounced at large *Da* (dynamic loading, Figure 8d) values. This leads to much greater fragmentation of the material (the size of the blocks is typically several times smaller than at a small *Da* value). The smaller characteristic size of fragments is due to the high pore pressure at large *Da* values, which leads to a significant increase in shear stresses in the vicinity of micropores and multiple fractures.

**Figure 9:**
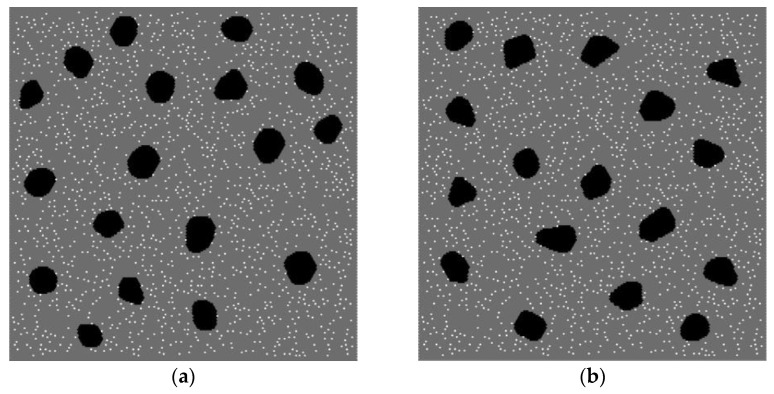
Examples of mesoscale water-saturated concrete samples with different spatial ar-rangements of aggregates and the same arrangement of micropores. Different initial values of the generator of random number were used to arrange aggregates in (**a**,**b**). The sample (**a**) is characterized by a slightly more uniform spatial distribution of aggregates and higher com-pressive strength in comparison with (**b**).

To study the effect of the spatial distribution of aggregates on the mechanical characteristics of mesoscale volumes, we generated five statistically equivalent model samples. All samples have the same arrangement of micropores in the cement stone (Figure 6b). Basalt aggregates were located in this matrix (in all cases, the volume concentration of aggregates was 10%). Different shapes, sizes, and spatial positions of the aggregates in different samples are due to different seed values of the generator of random numbers in the software generator of aggregates. Examples are shown in Figure 9.

The analysis of the simulation results shows that the values of local elastic parameters of concrete are almost independent of the spatial arrangement of aggregates and their geometric details. For example, Young’s modulus *E* is the same for all considered samples (the difference is less than 1%). The numerically obtained value *E* = 36.8 GPa agrees well with the estimate within the Reuss approximation of elastic moduli $1/E=C_{Cem}/E_{Cem}+\left(1-C_{Cem}\right)/E_{Bas}$ (*C_Cem_* is the volume fraction of porous mortar, *E* is Young’s modulus of the composite, *E_Cem_* and *E_Bas_* are Young’s moduli of porous mortar and basalt). The difference between the numerical and analytical estimates does not exceed 2%. This confirms that the considered samples are representative mesoscopic volume elements.

The approximating sigmoid functions for different concrete specimens are almost parallel to each other, similar to the above case of cement stone. The sigmoids below correspond to the “limiting” cases (upper and lower boundary curves):

(22) $$\sigma_{comp}=\sigma_{comp}^{\min}+\frac{\sigma_{comp}^{\max}-\sigma_{comp}^{\min}}{\left(1+{\left[Da/5.02\right]}^{1.82}\right)},$$

where $\sigma_{comp}^{\min}$ ≈ 47.26 MPa and $\sigma_{comp}^{\max}$ ≈ 53.44 MPa;

(23) $$\sigma_{comp}=\sigma_{comp}^{\min}+\frac{\sigma_{comp}^{\max}-\sigma_{comp}^{\min}}{\left(1+{\left[Da/3.13\right]}^{1.86}\right)},$$

where $\sigma_{comp}^{\min}$ ≈ 42.71 MPa and $\sigma_{comp}^{\max}$ ≈ 47.37 MPa.

**Figure 10:**
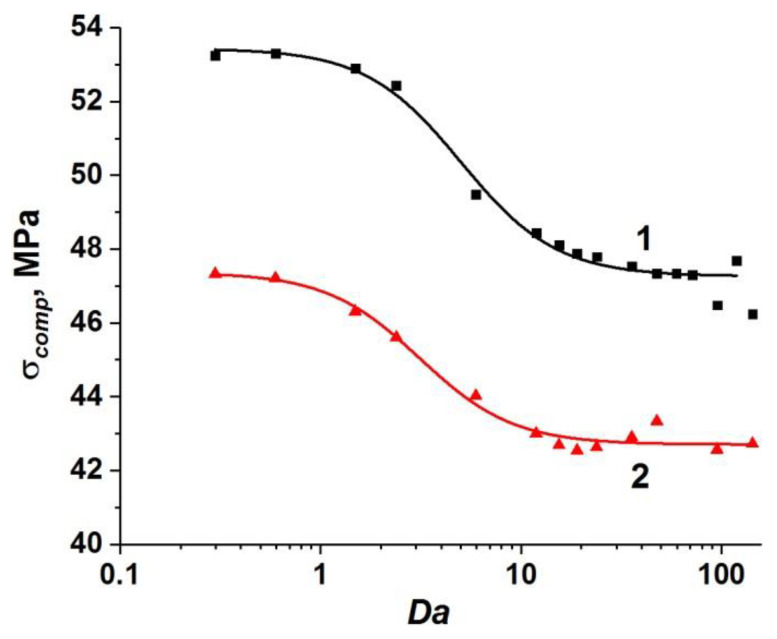
Dependence of the compressive strength of water-saturated concrete samples with the same microporous structure but different spatial arrangements of aggregates. Curves 1 and 2 correspond to the samples in Figure 9a,b.

Functions (22) and (23) are shown in Figure 10. Curves σ*_comp_*(*Da*) for the rest of the mesoscale concrete samples lie within the interval bounded by these two “limiting” curves.

The width of the σ*_comp_* variation interval is about 10% of the strength of dry concrete and does not depend on the value of *Da* (in other words, it is the same in both quasi-static and dynamic regimes of loading). Note that the intervals of variation of other parameters of the approximating sigmoid are also moderate.

Summarized results of the numerical analysis of the effect of interstitial fluid on dynamic strength of mesoscopic representative volumes of high-strength concrete are shown in Table 4. The data in the table are based on the processing of 5 statistically equivalent mesoscale samples.

Therefore, the features of the spatial arrangement of micropores and hardening aggregates mainly determine the characteristic level of local strength and insignificantly affect the strain rate sensitivity of concrete. Both types of structural elements are strong mesoscale stress concentrators. Comparison of the relative contributions of micropores and aggregates to the value of the variation interval of the local dynamic strength shows that aggregates are the most significant factor. Their heterogeneous spatial distribution provides a 2–2.5 times wider range of variation in local strength than micropores in the cement stone.

## 4. Discussion

The simulation results showed that the redistribution (flow) of pore fluid in the pore space is a key factor controlling the systematic change of compressive strength and fracture pattern of concrete samples in the “intermediate” interval of strain rates (10^−4^ s^−1^ < $\dot{\varepsilon }$ < 10^1^ s^−1^). We should remember that this interval separates the quasi-static and high strain rate regions. Since the fluid flow rate is controlled by applied strain rate, the dependence of the mechanical parameters (including compressive strength) and fracture features of fluid-saturated concrete samples on the loading dynamics is adequately characterized by the general loading rate parameter (Darcy number). In other words, the control parameter is not simply the strain rate, but the balance of the strain and fluid flow rates.

The structural factor that has an important influence on the rate sensitivity of strength and fracture pattern of concrete is porosity. The considered high-strength concrete has two-scale porosity. Although the influence of capillary and microporosity on fracture and compressive strength is generally similar, there are significant differences between them.

The open capillary network determines the phenomenon of strain-rate sensitivity of fluid-saturated concrete and logistic form of the compressive strength dependence on the rate parameter (*Da*). An increase in *Da* is accompanied by an increase in pore pressure in cement stone. On the one hand, this determines an increase in the effective stiffness (bulk modulus) of the matrix. On the other hand, this leads to a decrease in the threshold of plasticity and strength. The increase in pore pressure determines the onset of fracture of the matrix at lower values of the applied load and, consequently, a decrease in the uniaxial compressive strength of the sample. At the same time, a decrease in the plasticity threshold and an increase in the effective stiffness of the fluid-saturated matrix lead to a change in the contributions of mesoscopic structural elements to fracture.

**Figure 11:**
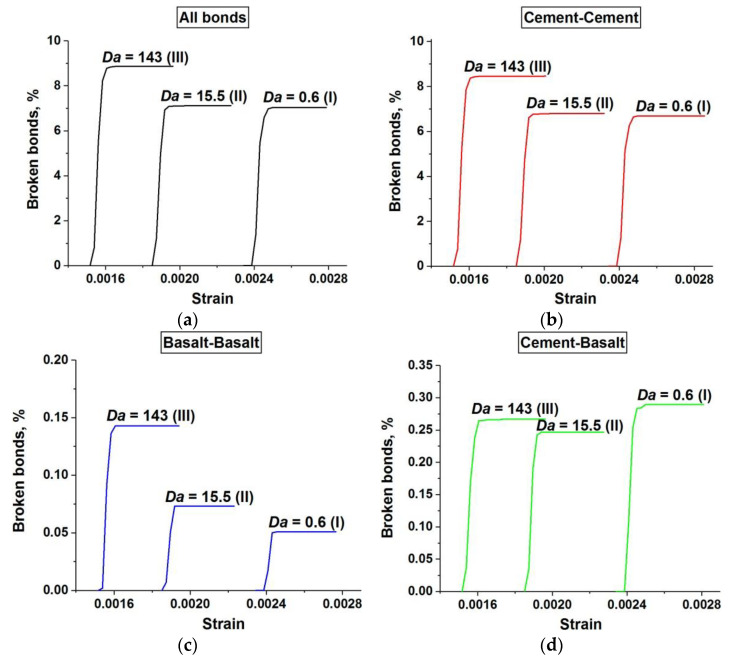
The dependences of the number of broken bonds in the fluid-saturated concrete sample with one-scale pore structure (capillary porosity) on the value of applied strain: (**a**) all broken bonds; (**b**) broken bonds in cement stone; (**c**) broken bonds in basalt aggregates; (**d**) broken bonds at the interphase boundaries. The number of broken bonds is normalized by the total number of bonds in the unstrained sample. Roman numerals I, II, and III denote the number of the corresponding section of the unified curve σ*_comp_*(*Da*).

These conclusions are confirmed by a quantitative analysis of the damage accumulation in concrete samples with a single-scale pore structure (only capillary pores). The term “damage” hereinafter means a broken bond between two adjacent discrete elements. Figure 11 shows the relationship between the number of broken bonds in the sample and the applied strain. The number of broken bonds is normalized by the total number of bonds in the unstrained sample. Four graphs depict the dynamics of the accumulation of all damages (a), damages in the cement stone matrix (b), damages in the basalt aggregates (c), and damages at the “matrix-aggregate” interfaces (d). Each graph shows three curves illustrating the dynamics of damage accumulation at different values of *Da* corresponding to the three main stages of the dependence σ*_comp_*(*Da*) (curve 1 in Figure 3).

First, we have to mention a systematic almost twofold decrease in the value of strain of the beginning of intensive damage accumulation with an increase in *Da*. This is a consequence of an increase in the characteristic pore pressure level. Second, an increase in Da is also accompanied by a moderate (about 30%) increase in the total number of damages in the sample (Figure 11a). This indicates a moderate increase in the degree of its fragmentation (Figure 4). This effect takes place mainly in the range of *Da* values corresponding to stage III of the dependence σ*_comp_*(*Da*) since the pore pressure in the matrix is close to maximum at this stage. The increase in damage is achieved primarily through matrix contribution (Figure 11b). At the same time, it is important to note a threefold increase in the contribution of aggregates to sample fracture with an increase in *Da* (Figure 11c) along with an almost unchanged contribution of interfaces (Figure 11d). This fact is clearly associated with an effective increase in the volumetric stiffness of the fluid-saturated matrix and stress transfer from matrix to aggregates. Therefore, although the general pattern of fracture does not change significantly, an increase in the generalized loading rate (*Da*) leads to a much more intensive contribution of the “aggregate” mechanism to the fracture of fluid-saturated concrete.

Insertion of micropores into the structure of water-saturated concrete leads to a change in the trend of the dependence of the sample damage (and the contributions of mesoscopic structural elements) on the loading rate. Large pores (micropores) are strong local stress concentrators and are deformed more strongly than capillary pores. “Extra” fluid passes from micropores into capillary pores during the loading and increases the pore pressure in the capillary pore system of surrounding regions of the matrix. This has implications for both the fracture and strain rate dependence of concrete strength.

**Figure 12:**
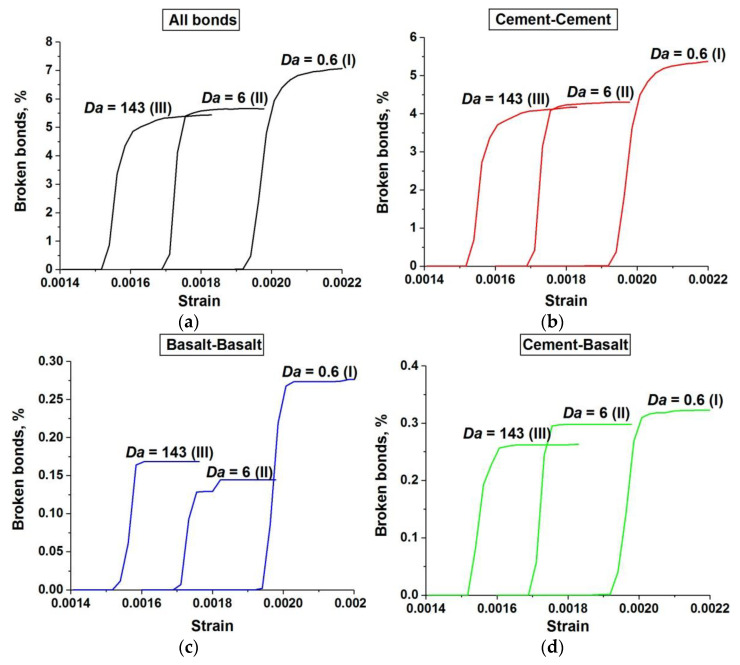
The dependences of the number of broken bonds in the fluid-saturated concrete sample with two-scale pore structure (capillary porosity and 4 vol.% micropores) on the value of applied strain: (**a**) all broken bonds; (**b**) broken bonds in cement stone; (**c**) broken bonds in basalt aggregates; (**d**) broken bonds at the interphase boundaries. The number of broken bonds is normalized by the total number of bonds in the unstrained sample. Roman numerals I, II, and III denote the number of the corresponding section of the unified curve σ*_comp_*(*Da*).

**Figure 13:**
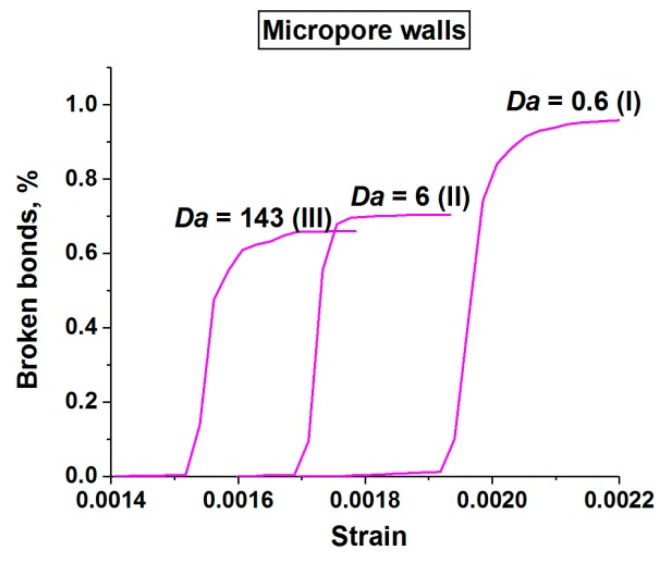
The dependence of the number of broken bonds in the walls of micropores in the fluid-saturated concrete sample with two-scale pore structure (capillary porosity and 4 vol.% micropores) on the value of applied strain. The number of broken bonds is normalized by the total number of bonds in the unstrained sample. Roman numerals I, II, and III denote the number of the corresponding section of the unified curve σ*_comp_*(*Da*).

First, an increase in *Da* is accompanied by an increase in the effective stiffness of micropores to a much greater extent than that of the surrounding cement stone matrix with capillary porosity. This leads to the fact that micropores become not voids, but inclusions with the stiffness of only an order of magnitude less than the stiffness of a cement stone (we can additionally mention that Poisson’s ratio of such an inclusion is 0.5). Hence, the concentration of deviatoric stresses in the matrix around micropores decreases, and the characteristic magnitude of compressive bulk stress increases. This effect provides a significant and gradual decrease in the total number of damages and contributions of all structural elements with an increase in *Da* (Figure 12). In other words, the fracture pattern of concrete with a two-scale pore structure becomes more localized compared to concrete with only a capillary pore network (Figure 11 and Figure 12). Moreover, the degree of fracture localization in fluid-saturated concrete with a two-scale pore structure increases with the loading rate (*Da*). This is achieved by reducing the number of individual damages and short internal cracks (the effect is especially pronounced for the aggregates, Figure 12c). The destruction is carried out by the development of a system of large cracks. These cracks are generated mainly on micropores. However, a dramatic increase in the effective stiffness of micropores with an increase in *Da* leads to the number of such centers of crack initiation being almost halved (Figure 13).

Second, some fraction of micropore fluid passes into capillary pores during the loading and additionally increases the pore pressure in the capillary pore system. Since micropores are deformed larger than capillary pores, the characteristic rate of fluid flow from micropores to the surrounding matrix is higher than inside the matrix. In addition, the characteristic distance between micropores is much less than the half-width of the sample. Therefore, the effect of pore fluid transport between micro- and capillary pore spaces on the stress state and strength of concrete is valuable already at *Da* corresponding to low strain rates. The consequence is an “earlier” transition from stage I to stage II (Figure 3), that is, the dynamic strength of concrete with micropores begins to decrease at significantly lower strain rates. The foregoing suggests that an increase in the number of scales of the pore structure of fluid-saturated concrete is accompanied by a decrease in the threshold of its strain rate sensitivity.

Summarizing the above, the fracture pattern and dynamic compressive strength of water-saturated concrete with two-scale porosity are determined not only by the parameters of the capillary pore system (mainly by permeability) but also by the volume fraction of large (micro-scale) pores. An increase in the fraction of micropores leads not only to a common decrease in the strength of the samples but also to a decrease in the threshold for the strain rate sensitivity of strength from *Da*~10^1^ down to *Da*~10^−1^ (Figure 3). For water-saturated samples with a transverse size (width) of 5–10 cm, this corresponds to a shift in the characteristic threshold values of the strain rate $\dot{\varepsilon }$ from 10^−2^–10^−1^ s^−1^ down to 10^−4^–10^−3^ s^−1^. The interval of significant change in strength also changes (expands) by two orders of magnitude. In terms of the parameters of the sigmoid function σ*_comp_*(*Da*), this is accompanied by a more than twofold decrease in the exponent and a shift of the median point of the sigmoid to the left by an order of magnitude.

**Figure 14:**
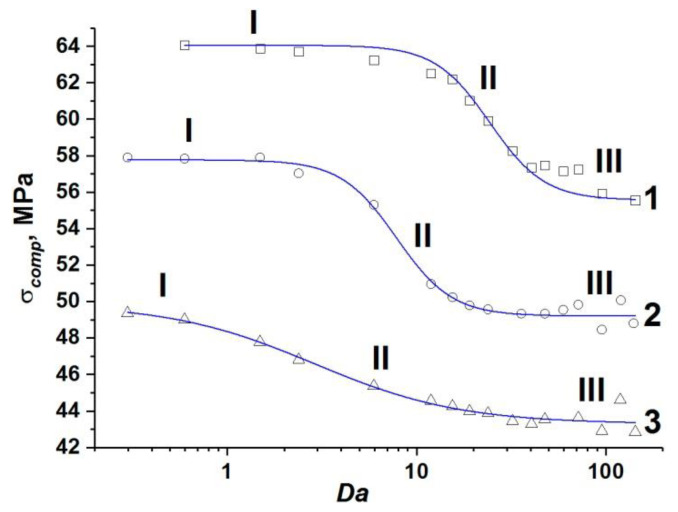
Dependence of the compressive strength of fluid-saturated mesoscale concrete samples with different volume fractions of micropores on the Darcy number *Da*: 0% (curve 1); 2% (curve 2); 4% (curve 3). Curves 1 and 3 correspond to curves 1 and 2 in Figure 3.

It is important to note that the change in the parameters of the unified curve σ*_comp_*(*Da*), with an increase in the volume fraction of micropores, is strongly nonlinear. This is illustrated in Figure 14, which shows σ*_comp_*(*Da*) curves for the samples with three different volume fractions of micropores (0%, 2%, and 4%). One can see that the σ*_comp_*(*Da*) curve shifts down and to the left almost without distortion at “small” values of volume fraction (the exponent of the sigmoid for micropore fraction 2% is the same as for 0%, while *Da* value of the midpoint of the sigmoid decreases three times). A further increase in the volume fraction of micropores is accompanied by the expansion of transitional section II at the expense of section I with an almost unchanged value of the median point of the sigmoid.

Such a staged nature of the change in the parameters of the dependence σ*_comp_*(*Da*) is explained by the mutual influence of micropores. At low concentrations of micropores, the distance between them is significantly higher than the size of the region of the surrounding matrix, in which the pore pressure significantly increases due to fluid transport from the micropore. A further increase in the volume fraction of micropores (i.e., a decrease in the characteristic distance between micropores) leads to the influence of micropores on the pore pressure and the dynamics of the fluid flow process throughout the cement stone matrix.

Therefore, the mechanical effect of pore pressure on the compressive strength of concretes with a two-scale pore structure (strength reduction) can take place already at low strain rates approaching the quasi-static loading regime. The factor controlling the threshold (minimum) values of $\dot{\varepsilon }$ is microporosity (the volume fraction of micropores must exceed some characteristic value of the order of several percent), while the capillary network determines the phenomenon of strain-rate sensitivity of the concrete and logistic form of the dependence of compressive strength on strain rate.

## 5. Conclusions

The paper numerically studied the mechanical effect of pore fluid pressure on the fracture and dynamic strength of high-strength concrete under uniaxial compression. We considered the “intermediate” range of strain rates between quasi-static (<10^−4^ s^−1^) and large (≥10^0^ s^−1^) values. The peculiarities of this interval are: (1) insignificant strain rate sensitivity of the mechanical characteristics of dry concrete; (2) the determining role of competition between pore pressure and pore fluid flow in the mechanical effect of interstitial fluid on the stresses and strength of concrete.

The study was carried out with the use of a previously developed coupled numerical (discrete element) model of high-strength concrete with two-scale porosity and interstitial fluid [65]. In the previous paper [65], we first showed that sensitivity of the compressive strength of fluid-saturated concrete to loading rate can be effectively characterized by the Darcy number (*Da*). Parameter *Da* is a measure of the balance of pore space deformation and pore fluid flow and a dimensionless generalization of the traditionally used strain rate to the fluid-saturated materials. We revealed the nonlinear (logistic) decreasing dependence of the compressive strength on Darcy number and showed that the parameters of this dependence are different for concretes with one-scale and two-scale porosities.

In the present study, we comprehensively investigated how the capillary and micropore subsystems determine the change in the fracture pattern and dynamic compressive strength of fluid-saturated high-strength concrete with an increase in the loading rate characterized by the *Da* value. The following observations and conclusions can be dawn:

- The effect of pore fluid on the dynamic mechanical behavior of concrete is determined not only by the absolute value of the open porosity but also by the number of pore scales. We showed that fracture of concrete with only low-scale porosity (capillary pore network) is typically brittle and is characterized by the formation of a network of internal “secondary” cracks. It is important to emphasize the formation of regions with the strong breaking of the cement stone matrix (cleavage zones) up to the crushing of the material. The introduction of large-scale pores (micropores) leads to a qualitative change in the dynamics of crack growth and a strongly pronounced localization of fracture. Cracks originate not at interphase boundaries (as with one-scale porosity) but at micropores and develop in a jump-like manner (from micropore to micropore). Moreover, there are no cleavage zones even at sufficiently high *Da* values corresponding to characteristic strain rates >1 s^−1^. The latter is a consequence of the large number of centers of crack nucleation (micropores), which allow efficient relaxation of high local shear stresses without cleavage and crushing of the material.
- Large pores (micropores) also determine a qualitative change in the fracture trend with an increase in the loading rate (characterized by the value of *Da*). An increase in *Da* value leads to an increase in “multiplicity” of fracture of the concrete with only low-scale (capillary) porosity. The number of main cracks and connecting internal cracks in the matrix increases with the value of *Da*. The sizes of cleavage zones and their number increase in multiples. This can be generally characterized by an increase in the total damage of concrete (the amount of mesoscale damages increases). The opposite trend takes place for concrete with low-scale and large (microscopic) pores. The total damage of the samples systematically decreases with an increase in *Da* due to a decrease in the number of single mesoscopic damages and short internal cracks. Fractures develop by means of the formation of large cracks. We also emphasize the systematic change in the contribution of aggregates to fracture in both systems with an increase in *Da* value. At a small *Da* value, the cracks predominantly bend around hard aggregates through interfaces. At large *Da* values, many cracks cut aggregates (the fraction of fractured particles increases with an increase in *Da*).
- The above trends in the change of fracture with an increase in *Da* are determined by the increasing characteristic value of the pore fluid pressure and its contribution to the internal stresses in the solid-phase skeleton of cement stone. At the same time, the effects of this contribution differ for concretes with one-scale (capillary) and two-scale (capillary and micropores) porosity. The increase in pore pressure determines the onset of fracture of the matrix at lower values of the applied stress. In the first case (only low scale porosity), this determines an increase in damage of the mesoscopically homogeneous matrix material with an increase in *Da*. The micropores with interstitial fluid provide the mesoscopic heterogeneity of the matrix and the corresponding inhomogeneity of stress distribution. Micropores deform more strongly than capillary pores in the matrix, and the pore pressure in the micropore grows faster than in the surrounding region of the matrix. An increase in *Da* is accompanied by an increase in effective stiffness of these stress mesoconcentrators due to the finite rate of fluid outflow to the capillary network. Hence, the concentration of deviator stress in the matrix around micropores decreases, while the characteristic magnitude of compressive volumetric stress increases. This effect provides the above described significant and gradual decrease in the total number of damages with an increase in *Da* value.
- The above results allow us to conclude that expanding the range of scales of the pore structure contributes to an increase in the localization of fracture of water-saturated concrete and to a decrease in the degree of fragmentation under compression. This conclusion can be useful for the design of the internal structure of concrete, which must meet special requirements for fracture.
- Micropores reduce not only the overall compressive strength of concrete (by ~25%) but also the threshold of the sensitivity of concrete to the loading rate (by two orders of magnitude). A noticeable decrease in the strength of concrete with a two-scale pore structure begins at *Da*~10^−1^, while the sensitivity threshold for concrete with only a capillary pore system is *Da* ≈ 10. Moreover, the length of the *Da* interval corresponding to a decrease in concrete strength from drained (maximum) to undrained (minimum) values increases by two orders of magnitude (the exponent of the approximating sigmoid is almost halved). This is a consequence of the above-described effect of a stronger increase in pore pressure in micropores during compression. Indeed, the pore pressure gradient between the micropore and the surrounding matrix is higher than inside the matrix, and the characteristic rate of fluid flow from micropores to the surrounding matrix is higher than in the matrix. An intense inflow of fluid into the matrix accelerates the growth of pore pressure and the redistribution of fluid in the matrix. Therefore, the effect of pore fluid transport between micro- and capillary pore spaces on the stress state and strength of concrete is valuable already at *Da* values corresponding to low strain rates.
- The described effect of increasing the sensitivity of concrete to the loading rate depends on the characteristic distance between micropores (that is, on their concentration). At low concentrations, the distance between micropores is significantly larger than the characteristic size of the region of the surrounding matrix with increased pore pressure provided by fluid transport from the micropore. In this case, the decrease in the concrete sensitivity threshold is not accompanied by a noticeable change in the width of the “transition” interval of *Da* between the intervals corresponding to the maximum (drained) and the minimum (undrained) values of strength. An increase in the concentration of micropores (i.e., a decrease in the characteristic distance between micropores) leads to the influence of micropores on the pore pressure and the dynamics of the fluid flow throughout the cement stone matrix. Overcoming the “percolation threshold” is accompanied by the expansion of the transition interval of *Da*.
- An important result of the study is the revealed “stability” of strain rate dependence of the dynamic compressive strength for representative mesoscopic concrete volumes characterized by different spatial arrangements of micropores and hardening inclusions. Small variation intervals of the parameters of the approximating sigmoid indicate that the results of modeling of representative volume elements on the mesoscale can be used to forecast the dynamic strength of macroscopic samples and structural elements (except the elements containing macroscopic defects).

The results of the present study make a valuable contribution to the understanding of the role of pore pressure in the onset of the limiting state of concretes as well as other brittle solids with a multi-scale pore structure. These results can be particularly useful for developing predictive models of degradation and abrasive wear of offshore concrete structures in the Arctic under multiple dynamic contact loading by sea ice and/or other floating objects. The results of the study can also be used when solving the problems of determining the “optimal” parameters of the internal structure of concretes that function under conditions of moisture saturation or in an aqueous environment.

## Figures and Tables

**Table 1:** Physical and mechanical characteristics of the constituents of the model high-strength concrete.

Constituent	ρ (kg/m^3^)	φ (%)	*G* (GPa)	*K* (GPa)	*K_s_* (GPa)
Cement stone	3500	6	17	62.5	125
Basalt	2950	0	60	166	166

The following symbols are used in the Table: ρ is density, φ is capillary porosity, *G* is shear modulus, *K* is the bulk modulus, *K_s_* is the bulk modulus of the nonporous material (material of the skeleton walls). Detailed information about the meaning of these parameters is provided in Section 2.3.

**Table 2:** Parameters of the plasticity model for cement stone.

Cohesion (MPa)	Internal Friction Coefficient	Strain-Hardening Coefficient (GPa)
18.5	0.6	14.1

Detailed information about the plasticity model for cement matrix and the meaning of these parameters is provided in Section 2.3.

**Table 3:** Strength parameters for concrete constituents and matrix-aggregate interface.

Constituent	UCS (MPa)	UTS (MPa)
Cement stone	120	30
Basalt	260	52
Cement-basalt interface	120	30

Detailed information about the criteria of local fractures is provided in Section 2.3.

**Table 4:** Parameters of the logistic dependence of the uniaxial compressive strength of high-strength concrete with a two-scale porosity (6 vol.% of capillary pores and 4 vol.% of micropores) on the generalized loading rate parameter *Da*.

φ (%)	σ*_drain_* (MPa)	RMSD σ*_drain_* (MPa)	σ*_undrain_* (MPa)	RMSD σ*_undrain_* (MPa)	Midpoint	RMSD Midpoint	Power	RMSD Power
0	55.9	1.5	49.4	1.4	6.2	1.6	1.86	0.05
10	50.3	3.1	44.4	2.5	3.7	1.2	1.83	0.03

The following symbols are used in the Table: φ is the volume fraction of aggregates, σ*_drain_* is the maximum value of compressive strength (drained condition at low *Da*), σ*_undrain_* is the minimum value of compressive strength (undrained condition at large *Da*), Midpoint and Power are the midpoint and the power of approximating sigmoid, and RMSD is the root-mean-square deviation of the corresponding parameter.
